# Parcerias para o Desenvolvimento Produtivo no Brasil: evidências empíricas sobre capacidade produtiva (2011-2024)

**DOI:** 10.1590/0102-311XPT023826

**Published:** 2026-08-31

**Authors:** Alexandra Albareda, Ricardo Lobato Torres

**Affiliations:** 1 Universidade Federal do Paraná, Curitiba, Brasil.

**Keywords:** Parcerias Público-Privadas, Capacidade Organizacional, Importação de Produtos, Complexo Econômico-Industrial da Saúde, Public-Private Sector Partnerships, Organizational Capacity, Importation of Products, Health Economic-Industrial Complex, Asociación entre el Sector Público-Privado, Capacidad Organizacional, Importación de Productos, Complejo Económico-Industrial de la Salud

## Abstract

As Parcerias para o Desenvolvimento Produtivo (PDPs) configuram um dos principais instrumentos da política industrial em saúde no Brasil, ao articular o poder de compra do Estado à transferência de tecnologia para laboratórios farmacêuticos oficiais (LFOs). Este artigo avaliou em que medida as PDPs contribuíram para o fortalecimento da capacidade produtiva nacional de medicamentos estratégicos no período de 2011 a 2024. Realizou-se uma avaliação formativa de política pública, baseada em análise documental, dados de aquisições governamentais e construção de indicadores de sustentabilidade produtiva e tecnológica. Os resultados evidenciam avanços relevantes, porém desiguais, entre plataformas tecnológicas, instituições públicas e classes terapêuticas. As PDPs associadas a medicamentos sintéticos apresentaram maior estabilidade produtiva, já as parcerias em plataformas biotecnológicas enfrentaram maiores desafios de internalização tecnológica. Observou-se elevada heterogeneidade institucional e concentração de capacidades em poucos laboratórios públicos. Conclui-se que as PDPs representam um instrumento estratégico para o fortalecimento do Complexo Econômico-Industrial da Saúde, embora sua efetividade permaneça condicionada à superação de fragilidades estruturais e à consolidação de capacidades produtivas e tecnológicas de longo prazo.

## Abstract (en)

The Productive Development Partnerships (PDPs) are one of the main instruments of industrial health policy in Brazil, by articulating the purchasing power of the State with the transfer of technology to official pharmaceutical laboratories (LFOs). This article evaluated the extent to which PDPs contributed to strengthening the national production capacity of strategic medicines in the period from 2011 to 2024. A formative evaluation of public policy was carried out, based on documentary analysis, data from government acquisitions and the construction of indicators of productive and technological sustainability. The results show relevant, but uneven, advances between technological platforms, public institutions and therapeutic classes. PDPs associated with synthetic drugs showed greater production stability, while partnerships in biotechnology platforms faced greater challenges of technological internalization. High institutional heterogeneity and concentration of capacities were observed in a few public laboratories. It is concluded that the PDPs represent a strategic instrument for strengthening the Economic-Industrial Health Complex, although their effectiveness remains conditioned to the overcoming of structural weaknesses and the consolidation of long-term productive and technological capacities.

## Abstract (es)

Las Asociaciones para el Desarrollo Productivo (PDP) constituyen uno de los principales instrumentos de la política industrial en salud en Brasil, al articular el poder de compra del Estado con la transferencia de tecnología a los laboratorios farmacéuticos oficiales (LFO). Este artículo evaluó en qué medida las PDP contribuyeron a fortalecer la capacidad productiva nacional de medicamentos estratégicos durante el período comprendido entre 2011 y 2024. Se realizó una evaluación formativa de política pública, basada en análisis documental, datos de adquisiciones gubernamentales y construcción de indicadores de sostenibilidad productiva y tecnológica. Los resultados demuestran avances significativos, aunque desiguales, entre plataformas tecnológicas, instituciones públicas y clases terapéuticas. Las PDP asociadas a medicamentos sintéticos demostraron una mayor estabilidad productiva, mientras que las alianzas en plataformas biotecnológicas enfrentaron mayores desafíos en la internalización tecnológica. Se observó una elevada heterogeneidad institucional y concentración de capacidades en pocos laboratorios públicos. Se concluye que las PDP representan un instrumento estratégico para fortalecer el Complejo Económico-Industrial de la Salud, aunque su eficacia sigue dependiendo de la superación de debilidades estructurales y la consolidación de capacidades productivas y tecnológicas a largo plazo.

## Introdução

A garantia do acesso universal a medicamentos essenciais constitui um dos pilares dos sistemas públicos de saúde e depende, de forma crescente, da existência de capacidades produtivas nacionais capazes de assegurar o fornecimento contínuo e sustentável. Em países de industrialização tardia, a elevada dependência de importações de medicamentos, vacinas e insumos farmacêuticos ativos (IFAs) configura um risco econômico, sanitário e estratégico, ao expor os sistemas de saúde a vulnerabilidades associadas às cadeias globais de suprimento e às assimetrias tecnológicas internacionais ^1^.

No Brasil, essa vulnerabilidade assume contornos específicos em razão do desenho constitucional do Sistema Único de Saúde (SUS), que atribui ao Estado a responsabilidade pela garantia do direito à saúde em um sistema universal. A sustentabilidade do SUS, portanto, exige uma articulação estruturada entre políticas de saúde, políticas industriais e políticas de ciência, tecnologia e inovação, capazes de reduzir a dependência externa e ampliar a autonomia sanitária nacional. Nesse contexto, consolida-se o conceito de Complexo Econômico-Industrial da Saúde (CEIS) dentro do Governo Federal, que reconhece a saúde como setor estratégico de desenvolvimento e explicita a interdependência entre produção, inovação tecnológica e garantia de direitos sociais ^2^ ^,^ ^3^.

As Parcerias para o Desenvolvimento Produtivo (PDPs) constituem um dos principais instrumentos dessa estratégia. Instituídas a partir de 2008 e regulamentadas em 2012 e 2014, as PDPs foram concebidas como mecanismo de articulação entre o poder de compra do Estado e a transferência de tecnologia do setor privado para laboratórios farmacêuticos oficiais (LFOs), com o objetivo de internalizar a produção de medicamentos estratégicos para o SUS e fortalecer a base produtiva nacional ^4^ ^,^ ^5^. A literatura aponta avanços associados à redução de preços, à ampliação do acesso e à consolidação de parcerias público-privadas no setor farmacêutico ^6^ ^,^ ^7^.

Entretanto, estudos anteriores têm apontado alguns limites da política, como dificuldades na internalização plena das tecnologias, persistência da dependência de IFAs importados, heterogeneidade das capacidades institucionais dos laboratórios públicos e fragilidades nos mecanismos de governança e avaliação das parcerias ^8^ ^,^ ^9^ ^,^ ^10^. Ademais, ainda são escassas as análises empíricas de médio e longo prazo que avaliem os efeitos estruturais das PDPs sobre a capacidade produtiva nacional e as diferenças de desempenho entre plataformas tecnológicas, especialmente entre medicamentos sintéticos e biológicos ^3^ ^,^ ^11^ ^,^ ^12^ ^,^ ^13^.

Este artigo busca contribuir para esse debate ao avaliar empiricamente os resultados das PDPs no Brasil no período de 2011 a 2024, com foco nas fases III e IV das parcerias. Ao articular a análise de dados de aquisições governamentais e indicadores de sustentabilidade produtiva e tecnológica elaborados no trabalho, o estudo procura responder à seguinte questão central: em que medida as PDPs contribuíram para o fortalecimento da capacidade produtiva nacional de medicamentos estratégicos para o SUS?

Nesse sentido, o artigo tem como objetivo específico construir indicadores de sustentabilidade produtiva e tecnológica das PDPs, estruturados nas dimensões de continuidade produtiva, maturação tecnológica, escala/economicidade e autonomia produtiva relativa, com base na sistematização de dados administrativos, normativos e produtivos das parcerias, com ênfase nas fases III e IV. Essa estratégia analítica permite avaliar, de forma longitudinal e comparativa, os efeitos das PDPs sobre a capacidade produtiva nacional em medicamentos estratégicos para o SUS.

## Métodos

O estudo adotou uma abordagem de avaliação formativa de política pública, orientada à análise de resultados intermediários e estruturais das PDPs. Essa opção metodológica é adequada por se tratar de uma política em execução durante a maior parte do período analisado, cujos efeitos mais relevantes - como a internalização tecnológica e a consolidação produtiva - manifestam-se de forma progressiva, não linear e condicionada por fatores institucionais e estruturais ^11^ ^,^ ^12^.

A avaliação formativa permite identificar padrões de desempenho, gargalos e limitações ainda durante a execução da política, diferindo de abordagens exclusivamente *ex post*, que tendem a capturar apenas resultados finais e, muitas vezes, desconsideram os processos de aprendizagem e adaptação institucional inerentes a políticas industriais e tecnológicas. Nesse sentido, a estratégia adotada buscou analisar as PDPs como instrumentos dinâmicos, sujeitos a ajustes normativos, variações conjunturais e assimetrias institucionais ao longo do tempo.

Foi realizado um estudo de natureza exploratória e analítico-descritiva, com base na triangulação de métodos quantitativos e qualitativos. O recorte temporal compreendeu o período de 2011 a 2024, escolhido por abranger as fases de consolidação normativa das PDPs, sua expansão inicial, os ciclos de instabilidade institucional e os estágios mais avançados de execução de parte das parcerias.

Foram incluídos na análise apenas medicamentos, vacinas e hemoderivados vinculados a PDPs que atingiram a fase III ou a fase IV até o ano de 2025, independentemente de estarem ativas, suspensas ou extintas no período analisado, o que resultou em 63 parcerias correspondentes a 45 medicamentos (visto que há casos de mais de uma parceria firmada para o mesmo produto).

Destaca-se que a fase III corresponde ao estágio em que inicia a transferência efetiva de tecnologia e a produção local com aquisição governamental, e a fase IV refere-se à verificação da internalização tecnológica e da capacidade produtiva do laboratório público ^5^. Essa delimitação permitiu concentrar a análise nos estágios em que se esperaria observar impactos sobre a capacidade produtiva nacional.

### Fontes de dados

A análise baseou-se em dois conjuntos principais de dados: (i) documentação normativa e administrativa do Ministério da Saúde - incluindo decretos, portarias, relatórios técnicos e documentos oficiais relacionados à formulação, regulamentação e monitoramento das PDPs, com destaque para as normativas publicadas a partir de 2012 e suas atualizações ^4^ ^,^ ^5^ ^,^ ^14^; (ii) dados de aquisições governamentais de medicamentos objeto das PDPs realizadas pelo Ministério da Saúde, contemplando volumes, valores contratados, fornecedores e períodos de compra. Esses dados permitiram analisar, de forma integrada, a evolução institucional da política e a continuidade do fornecimento ao SUS.

Com base nesse conjunto de informações, foram construídos indicadores de sustentabilidade produtiva e tecnológica das PDPs (Quadro 1), organizados em dimensões analíticas que dialogam com a literatura sobre o CEIS e sobre as políticas industriais em saúde ^1^ ^,^ ^2^. Os indicadores resultam de uma revisão sistematizada da literatura nacional sobre PDPs, política industrial e sustentabilidade produtiva, baseando-se na identificação de categorias analíticas recorrentes, métricas previamente utilizadas e lacunas empíricas ainda pouco exploradas. Parte dos indicadores foi inspirada diretamente em proposições existentes e outros foram adaptados ou desenvolvidos para atender às especificidades do contexto institucional brasileiro, dos medicamentos analisados e do recorte temporal adotado.

**Quadro 1:** Indicadores de sustentabilidade produtiva e tecnológica das Parcerias para o Desenvolvimento Produtivo (PDPs).

DIMENSÃO PRODUTIVA		
INDICADOR	FÓRMULA	DESCRIÇÃO
(1) Percentual de PDPs em fase IV (internalização produtiva)	(Nº de PDPs em fase IV ÷ Nº total de PDPs vigentes e suspensas) × 100	Reflete a taxa de internalização efetiva da produção nacional
(2) Tempo médio de transição da fase III → fase IV	Média aritmética (em anos) entre as datas de início das fases III e IV por TC (Quadro 07)	Indica o ritmo de maturação produtiva
(3) Taxa de extinção e suspensão de PDPs	ENT#091;(Nº de PDPs extintas + suspensas) ÷ Nº total de PDPs homologadasENT#093; × 100	Indica a estabilidade institucional e a robustez da política ao longo do tempo
(4) Percentual de PDPs por plataforma tecnológica	Distribuição percentual de sintéticos, biotecnológicos, vacinas, hemoderivados	Indica a concentração produtiva e a diversificação tecnológica
(5) Participação relativa de LFOs na fase IV	(Nº de PDPs fase IV por LFO ÷ total fase IV) × 100	Distribuição das PDPs internalizadas por laboratório
DIMENSÃO TECNOLÓGICA		
INDICADOR	FÓRMULA	DESCRIÇÃO/ACHADO PRINCIPAL
(6) Índice de complexidade tecnológica	ENT#091;(PDPs biotecnológicas + vacinas + hemoderivados) ÷ total de PDPsENT#093; × 100	Mede o grau de incorporação de tecnologias de fronteira
(7) Tempo médio de maturação tecnológica por plataforma	Média do tempo (anos) fase III → IV por plataforma (sintéticos, biotecnológicos etc.)	Indica o ritmo diferenciado de absorção tecnológica
(8) Taxa de sucesso tecnológico (fase III → fase IV)	(Nº de PDPs que alcançaram fase IV ÷ Nº de PDPs que chegaram à fase III) × 100	Mede a efetividade da transferência de tecnologia
(9) Percentual de produtos internalizados com produção contínua	(Nº de PDPs fase IV com vendas regulares ao Ministério da Saúde * ÷ total fase IV) × 100	Avalia a sustentabilidade tecnológica e a continuidade de oferta

LFO: laboratórios farmacêuticos oficiais; TC: Termos de Compromisso.

Fonte: elaboração própria.

* Consideraram-se regulares as vendas apontadas pelos dados econômicos de todas as PDPs ativas em fases III e IV realizadas entre 2020 e 2024, sendo consideradas não regulares as vendas anteriores a 2020.

As dimensões analíticas contempladas no Quadro 1 incluem: continuidade produtiva, associada à estabilidade do fornecimento e à previsibilidade da produção ^3^ ^,^ ^11^; escala produtiva, vinculada ao volume de produção, à capacidade instalada e ao uso do poder de compra do Estado ^2^ ^,^ ^7^; e economicidade, referente à eficiência relativa, aos custos e à racionalidade do gasto público nas compras governamentais em saúde ^6^ ^,^ ^11^. Esses referenciais orientaram a seleção e a operacionalização dos indicadores, assegurando coerência teórica e consistência metodológica.

As principais dimensões analíticas consideradas incluíram: (i) continuidade produtiva, expressa pela regularidade das aquisições governamentais; (ii) maturação tecnológica, associada ao avanço das PDPs entre as fases III e IV; e (iii) autonomia produtiva relativa, associada à dependência de fornecedores privados e à capacidade de atendimento da demanda pelo setor público. Esses indicadores permitiram análises comparativas entre PDPs, instituições públicas e plataformas tecnológicas, bem como a identificação de padrões agregados e assimetrias estruturais.

A análise incorporou um eixo comparativo entre medicamentos sintéticos e biológicos, considerando diferenças estruturais de complexidade produtiva, intensidade tecnológica e exigências regulatórias, reconhecidas na literatura como condicionantes das trajetórias de internalização tecnológica e da sustentabilidade produtiva das PDPs ^8^ ^,^ ^10^. Essa comparação possibilitou identificar padrões diferenciados de desempenho e limites específicos associados a cada plataforma.

Os dados quantitativos foram analisados por meio de estatística descritiva e análise de tendências temporais, com foco na identificação de padrões de continuidade, interrupção ou inflexão no período de 2011 a 2024, enquanto os dados qualitativos provenientes da análise documental foram utilizados de forma complementar. A triangulação entre fontes e métodos buscou aumentar a confiabilidade analítica, com análises conduzidas em nível agregado - examinando tendências gerais da política - e desagregado - focando medicamentos específicos, fases das PDPs e plataformas tecnológicas -, respeitando os limites de disponibilidade, qualidade e comparabilidade dos dados.

## Resultados

### Configuração geral das PDPs e distribuição institucional

Entre 2011 e 2024, as PDPs abrangeram um amplo conjunto de medicamentos, vacinas e hemoderivados estratégicos para o SUS. Em 2025, as parcerias estavam concentradas majoritariamente nas fases III (18 projetos) e IV (38 projetos) que, em conjunto, representavam 71,6% das PDPs vigentes. Em dezembro de 2024, havia ainda sete Termos de Compromisso suspensos, sendo cinco na fase II e dois na fase IV. Quanto à distribuição por plataforma tecnológica, identificaram-se 65 PDPs de medicamentos sintéticos, 17 de biotecnológicos, cinco de vacinas e uma de hemoderivados, evidenciando a predominância dos sintéticos ao longo de todo o período, ainda que com a incorporação gradual de parcerias biotecnológicas a partir da segunda metade da década de 2010.

Apesar da ampliação do escopo tecnológico, os dados indicam que tal movimento não resultou em maior diversificação institucional. A execução das PDPs permaneceu fortemente concentrada em poucos LFOs, com destaque para o Instituto de Tecnologia de Fármacos (Farmanguinhos) e o Instituto de Tecnologia em Imunobiológicos (Bio-Manguinhos), ambos da Fundação Oswaldo Cruz (Fiocruz), a Empresa Brasileira de Hemoderivados e Biotecnologia (Hemobrás) e o Instituto Butantan, que responderam por aproximadamente 80% dos valores contratados, cerca de 50% do volume de aquisições governamentais, e por aproximadamente três quartos das PDPs que alcançaram a fase IV entre as 56 parcerias que chegaram à fase III no período analisado. Essa concentração reflete diferenças históricas de capacidade produtiva, infraestrutura industrial, experiência regulatória e inserção institucional, favorecendo instituições com maior capacidade instalada e trajetória prévia na produção de medicamentos estratégicos.

Como as PDPs são dinâmicas e informações sobre mudanças de fases não estavam explícitas no *site* do Ministério da Saúde, foi realizado um requerimento de acesso à informação em maio de 2024, por meio da plataforma Fala.BR (https://falabr.cgu.gov.br), com retorno em julho do mesmo ano, questionando em que momento as PDPs haviam iniciado as fases III e IV. A informação de quais medicamentos estavam até então em fase III e fase IV e quando ocorreu essa transição pode ser conferida no Quadro 2. A análise das trajetórias entre as fases III e IV revela elevada heterogeneidade no desempenho das PDPs. Embora a fase III represente o início da transferência tecnológica e da produção local com aquisição governamental, e a fase IV a efetiva internalização tecnológica e autonomia produtiva, apenas uma parcela das parcerias avançou de forma consistente dentro dos prazos normativos. Em diversos casos, observou-se prolongamento da permanência na fase III, indicando dificuldades na conclusão da transferência tecnológica, validação de processos produtivos ou obtenção de certificações regulatórias.

**Quadro 2:** Início das fases III e IV das Parcerias para o Desenvolvimento Produtivo (PDPs) (apenas vigentes).

PDPs EM FASE III				
TERMO DE COMPROMISSO	PRODUTO	INSTITUIÇÃO PÚBLICA	INÍCIO DA FASE III	PREVISÃO DE INÍCIO DA FASE IV
TC nº 13/2017 TC nº 05/2018	Adalimumabe	Instituto Butantan	18/Nov/2022	18/Nov/2032
TC nº 19/2013 TC nº 03/2017	Adalimumabe	Fiocruz/Bio-Manguinhos	22/Jul/2022	22/Jul/2032
TC nº 11/2010	Betainterferona 1A	Fiocruz/Bio-Manguinhos	30/Nov/2015	30/Nov/2025
TC nº 17/2018	Darunavir	LAFEPE	09/Jan/2024	09/Jan/2029
TC nº 18/2018	Dolutegravir	LAFEPE	23/Nov/2021	23/Nov/2026
TC nº 18/2018	Dolutegravir	LAFEPE	23/Nov/2021	23/Nov/2026
TC nº 02/2012	Etanercepte	Fiocruz/Bio-Manguinhos	31/Mai/2019	31/Mai/2029
TC nº 09/2018	Everolimo	Fiocruz/Farmanguinhos	02/Abr/2024	02/Abr/2029
TC nº 29/2018	Everolimo	NUPLAM	11/Abr/2024	11/Abr/2029
TC nº 30/2018	Fingolimode	NUPLAM	24/Mar/2022	24/Mar/2027
TC nº 03/2018	Golimumabe	Fiocruz/Bio-Manguinhos	28/Set/2020	28/Set/2030
TC nº 03/2012	Rituximabe	Fiocruz/Bio-Manguinhos	28/Set/2020	28/Set/2030
TC nº 11/2018	Sofosbuvir	Fiocruz/Farmanguinhos	06/Nov/2023	06/Nov/2028
TC nº 13/2018	Sofosbuvir	FURP	12/Jan/2024	12/Jan/2029
TC nº 26/2013	Somatropina	Fiocruz/Bio-Manguinhos	30/Jun/2020	30/Jun/2030
TC nº 05/2012	Tenofovir + lamivudina (2 em 1)	LAFEPE	17/Mai/2021	17/Mai/2026
TC nº 05/2012	Tenofovir + lamivudina (2 em 1)	LAFEPE	17/Mai/2021	17/Mai/2026
TC nº 24/2013	Trastuzumabe	Fiocruz/Bio-Manguinhos	25/Set/2020	25/Set/2030
PDPs EM FASE IV				
TERMO DE COMPROMISSO	PRODUTO	INSTITUIÇÃO PÚBLICA	INÍCIO DA FASE III	INÍCIO DA FASE IV
TC nº 02/2011	Atazanavir	Fiocruz/Farmanguinhos	25/Jul/2014	25/Dez/2020
TC nº 08/2011	Cabergolina	Fiocruz/Farmanguinhos	07/Abr/2016	10/Jul/2021
TC nº 08/2009	Clozapina	LAFEPE	06/Jan/2011	06/Jan/2016
TC nº 05/2010	Entecavir	FUNED	13/Jun/2019	13/Jun/2024
TC nº 08/2018	Entricitabina + tenofovir	Fiocruz/Farmanguinhos	13/Dez/2019	13/Dez/2024
TC nº 08/2018	Entricitabina + tenofovir	Fiocruz/Farmanguinhos	13/Dez/2019	13/Dez/2024
TC nº 20/2012	Fator VIII recombinante	Hemobrás	16/Mai/2013	18/Ago/2023
TC nº 05/2013	Galantamina	FURP	09/Nov/2018	09/Nov/2023
TC nº 32/2013	Infliximabe	Fiocruz/Bio-Manguinhos	27/Nov/2014	27/Nov/2024
TC nº 09/2011	Leflunomida	LFM	15/Set/2014	15/Set/2019
TC nº 01/2012	Mesilato de imatinibe	Fiocruz/Farmanguinhos	22/Mar/2013	22/Mar/2018
TC nº 01/2012	Mesilato de imatinibe	IVB	22/Mar/2013	22/Mar/2018
TC nº 01/2012	Mesilato de imatinibe	IVB	22/Mar/2013	22/Mar/2018
TC nº 15/2012	Micofenolato de sódio	LQFEx	31/Mai/2016	17/Mar/2024
TC nº 27/2018	Micofenolato de sódio	LQFEx	31/Mai/2016	17/Mar/2024
TC nº 08/2009	Olanzapina	LAFEPE	19/Set/2012	19/Set/2017
TC nº 08/2009	Olanzapina	NUPLAM	28/Dez/2016	31/Dez/2023
TC nº 05/2011	Pramipexol	Fiocruz/Farmanguinhos	10/Out/2014	21/Jan/2021
TC nº 08/2009	Quetiapina	LAFEPE	19/Set/2012	19/Set/2017
TC nº 09/2009	Rifampicina + isoniazida + pirazinamida + etambutol (4 em 1 tuberculostático)	Fiocruz/Farmanguinhos	30/Nov/2015	15/Jul/2023
TC nº 06/2011	Riluzol	LFM	28/Dez/2015	28/Dez/2020
TC nº 07/2010	Ritonavir termoestável	LAFEPE	01/Ago/2017	09/Ago/2023
TC nº 01/2009	Rivastigmina	IVB	16/Ago/2012	16/Ago/2017
TC nº 01/2009	Rivastigmina	IVB	16/Ago/2012	16/Ago/2017
TC nº 07/2011	Sevelâmer	Fiocruz/Farmanguinhos	28/Dez/2016	30/Nov/2021
TC nº 36/2013	Sildenafila	LFM	26/Set/2019	26/Set/2024
TC nº 07/2009	Tacrolimo	Fiocruz/Farmanguinhos	16/Dez/2011	16/Dez/2016
TC nº 05/2009	Tenofovir	FUNED	13/Mai/2011	13/Mai/2016
TC nº 06/2009	Tenofovir	LAFEPE	22/Nov/2011	22/Nov/2016
TC nº 05/2012	Tenofovir + lamivudina (2 em 1)	Fiocruz/Farmanguinhos	13/Nov/2014	13/Nov/2019
TC nº 05/2012	Tenofovir + lamivudina (2 em 1)	Fiocruz/Farmanguinhos	13/Nov/2014	13/Nov/2019
TC nº 05/2012	Tenofovir + lamivudina (2 em 1)	Fiocruz/Farmanguinhos	13/Nov/2014	13/Nov/2019
TC nº 35/2013	Vacina DTPa (vacina adsorvida contra difteria, tétano e pertussis acelular)	Instituto Butantan	22/Jul/2016	22/Jul/2021
TC nº 09/2012	Vacina contra hepatite A	Instituto Butantan	10/Jan/2014	10/Jan/2020
TC nº 37/2013	Vacina contra HPV	Instituto Butantan	10/Jan/2014	10/Jan/2019
TC nº 09/2010	Ziprasidona	LFM	03/Mai/2017	03/Mai/2023

Bio-Manguinhos: Instituto de Tecnologia em Imunobiológicos; Farmanguinhos: Instituto de Tecnologia de Fármacos; Fiocruz: Fundação Oswaldo Cruz; FUNED: Fundação Ezequiel Dias; FURP: Fundação para o Remédio Popular; Hemobrás: Empresa Brasileira de Hemoderivados e Biotecnologia; IVB: Instituto Vital Brazil; LAFEPE: Laboratório Farmacêutico de Pernambuco; LFM: Laboratório Farmacêutico da Marinha; LQFEx: Laboratório Químico-Farmacêutico do Exército; NUPLAM: Núcleo de Pesquisa em Alimentos e Medicamentos da Universidade Federal do Rio Grande do Norte.

Fonte: elaboração própria com base nos dados disponibilizados pelo Ministério da Saúde (2024).

Nota: não há informação concreta sobre quando as parcerias extintas chegaram às fases III ou IV, sendo assim, as sete parcerias extintas analisadas não entraram nesta tabela.

Adicionalmente, identificou-se um número expressivo de PDPs que, apesar de terem alcançado a fase III, foram posteriormente suspensas ou extintas sem atingir a fase IV. Entre 2014 e dezembro de 2024, contabilizaram-se 66 parcerias extintas por motivos como inviabilidade econômica, desistência das instituições parceiras ou do Ministério da Saúde e descumprimento das condições aprovadas. Essas descontinuidades fragilizaram os resultados agregados da política ao interromper processos de aprendizagem tecnológica e comprometer investimentos realizados ao longo das parcerias.

Ainda, realizou-se uma análise das aquisições governamentais associadas às PDPs, o que revelou padrões distintos de comportamento ao longo do período analisado. A Tabela 1 apresenta os produtos adquiridos durante as PDPs, a sua plataforma (sintético, biotecnológico, hemoderivado ou vacina), o ano de aquisição, quantidades e valores totais. Levando-se em conta o extenso período analisado e a relevância de se obter dados reais para garantir a comparabilidade, os preços coletados na pesquisa foram ajustados usando-se o Índice Nacional de Preços ao Consumidor Amplo (IPCA), com dezembro de 2024 como período base.

**Tabela 1:** Valores pagos por produto, por ano, corrigidos pelo Índice Nacional de Preços ao Consumidor Amplo (IPCA) (ano-base 2024).

Produto	Instituição pública	Plataforma	Ano de publicação	Quantidade adquirida	Valor total (R$)	IPCA	Valor corrigido (R$)
Adalimumabe	Instituto Butantan	Biotecnológico	2022	398.318	166.098.606,00	1,1524	191.412.033,55
Adalimumabe	Fiocruz/Bio-Manguinhos	Biotecnológico	2022	531.090	233.610.558,30	1,1524	269.212.807,38
Adalimumabe	Fiocruz/Bio-Manguinhos	Biotecnológico	2024	623.026	264.669.668,74	1,0483	277.453.213,74
Adalimumabe	Fiocruz/Bio-Manguinhos	Biotecnológico	2024	724.484	303.283.492,08	1,0483	317.932.084,75
Alfataliglicerase	Fiocruz/Bio-Manguinhos	Biotecnológico	2013	14.602	13.313.903,16	1,7011	22.648.280,67
Alfataliglicerase	Fiocruz/Bio-Manguinhos	Biotecnológico	2014	15.360	14.592.000,00	1,642	23.960.064,00
Alfataliglicerase	Fiocruz/Bio-Manguinhos	Biotecnológico	2016	13.680	21.723.840,00	1,4712	31.960.113,41
Alfataliglicerase	Fiocruz/Bio-Manguinhos	Biotecnológico	2016	121.569	85.098.300,00	1,4712	125.196.618,96
Atazanavir	Fiocruz/Farmanguinhos	Sintético	2014	4.584.840	16.780.514,40	1,642	27.553.604,64
Atazanavir	Fiocruz/Farmanguinhos	Sintético	2014	6.000.000	19.980.000,00	1,642	32.807.160,00
Atazanavir	Fiocruz/Farmanguinhos	Sintético	2014	11.939.070	71.873.201,40	1,642	118.015.796,70
Atazanavir	Fiocruz/Farmanguinhos	Sintético	2014	22.500.000	122.850.000,00	1,642	201.719.700,00
Atazanavir	Fiocruz/Farmanguinhos	Sintético	2015	4.629.420	15.415.968,60	1,5779	24.324.856,85
Atazanavir	Fiocruz/Farmanguinhos	Sintético	2015	27.721.740	151.360.700,40	1,5779	238.832.049,16
Atazanavir	Fiocruz/Farmanguinhos	Sintético	2019	3.000.000	14.541.000,00	1,3413	19.503.843,30
Atazanavir	Fiocruz/Farmanguinhos	Sintético	2020	33.450.000	162.132.150,00	1,2982	210.479.957,13
Betainterferona 1A	Fiocruz/Bio-Manguinhos	Biotecnológico	2015	150.240	20.172.724,80	1,5779	31.830.542,46
Betainterferona 1A	Fiocruz/Bio-Manguinhos	Biotecnológico	2015	422.292	64.019.467,20	1,5779	101.016.317,29
Betainterferona 1A	Fiocruz/Bio-Manguinhos	Biotecnológico	2016	179.280	23.589.662,40	1,4712	34.705.111,32
Betainterferona 1A	Fiocruz/Bio-Manguinhos	Biotecnológico	2016	433.464	64.399.746,48	1,4712	94.744.907,02
Betainterferona 1A	Fiocruz/Bio-Manguinhos	Biotecnológico	2017	132.384	16.767.757,44	1,4083	23.614.032,80
Betainterferona 1A	Fiocruz/Bio-Manguinhos	Biotecnológico	2017	405.168	57.939.024,00	1,4083	81.595.527,50
Betainterferona 1A	Fiocruz/Bio-Manguinhos	Biotecnológico	2019	68.520	8.504.702,40	1,3413	11.407.357,33
Betainterferona 1A	Fiocruz/Bio-Manguinhos	Biotecnológico	2019	407.616	57.123.306,24	1,3413	76.619.490,66
Betainterferona 1A	Fiocruz/Bio-Manguinhos	Biotecnológico	2020	102.504	12.332.256,00	1,2982	16.009.734,74
Betainterferona 1A	Fiocruz/Bio-Manguinhos	Biotecnológico	2020	444.336	60.358.602,00	1,2982	78.357.537,12
Betainterferona 1A	Fiocruz/Bio-Manguinhos	Biotecnológico	2021	101.100	18.523.542,00	1,253	23.209.998,13
Betainterferona 1A	Fiocruz/Bio-Manguinhos	Biotecnológico	2021	257.520	52.997.616,00	1,253	66.406.012,85
Betainterferona 1A	Fiocruz/Bio-Manguinhos	Biotecnológico	2022	81.588	15.014.639,64	1,1524	17.302.870,72
Betainterferona 1A	Fiocruz/Bio-Manguinhos	Biotecnológico	2022	343.692	71.044.573,32	1,1524	81.871.766,29
Betainterferona 1A	Fiocruz/Bio-Manguinhos	Biotecnológico	2023	98.940	17.769.752,62	1,0945	19.448.994,24
Betainterferona 1A	Fiocruz/Bio-Manguinhos	Biotecnológico	2023	325.620	65.689.113,51	1,0945	71.896.734,74
Cabergolina	Bahiafarma	Sintético	2015	2.054.896	17.466.616,00	1,5779	27.560.573,39
Cabergolina	Bahiafarma	Sintético	2016	1.261.624	10.187.613,80	1,4712	14.988.017,42
Cabergolina	Fiocruz/Farmanguinhos	Sintético	2016	1.261.624	10.187.613,80	1,4712	14.988.017,42
Cabergolina	Fiocruz/Farmanguinhos	Sintético	2016	1.330.808	10.207.297,00	1,4712	15.016.975,35
Cabergolina	Bahiafarma	Sintético	2017	1.330.808	10.207.297,36	1,4083	14.374.936,87
Cabergolina	Fiocruz/Farmanguinhos	Sintético	2020	5.032.400	8.504.756,00	1,2982	11.040.874,24
Clozapina	LAFEPE	Sintético	2011	12.287.368	21.257.146,64	1,8245	38.783.664,04
Clozapina	LAFEPE	Sintético	2011	20.700.000	33.948.000,00	1,8245	61.938.126,00
Clozapina	LAFEPE	Sintético	2011	412.220	164.888,00	1,8245	300.838,16
Clozapina	LAFEPE	Sintético	2011	1.012.300	384.674,00	1,8245	701.837,71
Clozapina	LAFEPE	Sintético	2013	21.271.080	33.140.342,64	1,7011	56.375.036,86
Clozapina	LAFEPE	Sintético	2013	612.900	221.256,90	1,7011	376.380,11
Clozapina	LAFEPE	Sintético	2014	21.863.520	32.358.009,60	1,642	53.131.851,76
Clozapina	LAFEPE	Sintético	2014	1.042.860	357.700,98	1,642	587.345,01
Clozapina	LAFEPE	Sintético	2015	25.919.100	36.286.740,00	1,5779	57.256.847,05
Clozapina	LAFEPE	Sintético	2015	770.280	246.489,60	1,5779	388.935,94
Darunavir	LAFEPE	Sintético	2024	32.393.400	325.877.905,80	1,0483	341.617.808,65
Dolutegravir	LAFEPE	Sintético	2021	79.500.000	325.950.000,00	1,253	408.415.350,00
Entecavir	FUNED	Sintético	2019	5.610.900	47.468.214,00	1,3413	63.669.115,44
Entecavir	FUNED	Sintético	2021	5.500.000	49.665.000,00	1,253	62.230.245,00
Entecavir	FUNED	Sintético	2022	1.375.000	12.416.250,00	1,1524	14.308.486,50
Entecavir	FUNED	Sintético	2022	4.065.000	37.804.500,00	1,1524	43.565.905,80
Entricitabina + tenofovir	Fiocruz/Farmanguinhos	Sintético	2019	2.205.000	4.961.250,00	1,3413	6.654.524,63
Entricitabina + tenofovir	Fiocruz/Farmanguinhos	Sintético	2020	6.000.000	13.500.000,00	1,2982	17.525.700,00
Entricitabina + tenofovir	Fiocruz/Farmanguinhos	Sintético	2021	14.900.000	40.826.000,00	1,253	51.154.978,00
Entricitabina + tenofovir	Fiocruz/Farmanguinhos	Sintético	2022	16.275.000	52.242.750,00	1,1524	60.204.545,10
Entricitabina + tenofovir	Fiocruz/Farmanguinhos	Sintético	2023	18.300.000	58.743.000,00	1,0945	64.294.213,50
Entricitabina + tenofovir	Fiocruz/Farmanguinhos	Sintético	2023	18.300.000	58.743.000,00	1,0945	64.294.213,50
Entricitabina + tenofovir	Fiocruz/Farmanguinhos	Sintético	2024	36.009.990	109.830.469,50	1,0483	115.135.281,18
Etanercepte	Fiocruz/Bio-Manguinhos	Biotecnológico	2019	688.000	200.345.600,00	1,3413	268.723.553,28
Etanercepte	Fiocruz/Bio-Manguinhos	Biotecnológico	2020	1.053.332	293.879.628,00	1,2982	381.514.533,07
Etanercepte	Fiocruz/Bio-Manguinhos	Biotecnológico	2021	506.408	153.208.676,00	1,253	191.970.471,03
Etanercepte	Fiocruz/Bio-Manguinhos	Biotecnológico	2022	592.188	184.336.280,64	1,1524	212.429.129,81
Etanercepte	Fiocruz/Bio-Manguinhos	Biotecnológico	2023	571.064	172.250.205,64	1,0945	188.527.850,07
Etanercepte	Fiocruz/Bio-Manguinhos	Biotecnológico	2024	55.000	16.348.200,00	1,0483	17.137.818,06
Everolimo	FURP	Sintético	2014	1.269.540	8.163.142,00	1,642	13.403.879,16
Everolimo	FURP	Sintético	2014	1.108.140	10.693.551,00	1,642	17.558.810,74
Everolimo	FURP	Sintético	2014	2.348.580	30.202.739,00	1,642	49.592.897,44
Everolimo	FURP	Sintético	2015	317.340	2.040.972,21	1,5779	3.220.450,05
Everolimo	FURP	Sintético	2015	277.020	2.673.797,04	1,5779	4.218.984,35
Everolimo	FURP	Sintético	2015	587.100	7.551.867,30	1,5779	11.916.091,41
Everolimo	Fiocruz/Farmanguinhos	Sintético	2024	1.085.280	5.068.257,60	1,0483	5.313.054,44
Everolimo	Fiocruz/Farmanguinhos	Sintético	2024	486.960	3.530.460,00	1,0483	3.700.981,22
Everolimo	Fiocruz/Farmanguinhos	Sintético	2024	1.224.000	11.848.320,00	1,0483	12.420.593,86
Everolimo	NUPLAM	Sintético	2024	1.085.280	5.068.257,60	1,0483	5.313.054,44
Everolimo	NUPLAM	Sintético	2024	486.960	3.530.460,00	1,0483	3.700.981,22
Everolimo	NUPLAM	Sintético	2024	1.224.000	11.848.320,00	1,0483	12.420.593,86
Fator VIII recombinante	Hemobrás	Hemoderivado	2013	350.000.000	257.250.000,00	1,7011	437.607.975,00
Fator VIII recombinante	Hemobrás	Hemoderivado	2014	87.500.000	64.312.500,00	1,642	105.601.125,00
Fator VIII recombinante	Hemobrás	Hemoderivado	2015	420.000.000	352.800.000,00	1,5779	556.683.120,00
Fator VIII recombinante	Hemobrás	Hemoderivado	2015	30.000.000	25.200.000,00	1,5779	39.763.080,00
Fator VIII recombinante	Hemobrás	Hemoderivado	2016	75.000.000	63.000.000,00	1,4712	92.685.600,00
Fator VIII recombinante	Hemobrás	Hemoderivado	2016	410.000.000	471.500.000,00	1,4712	693.670.800,00
Fator VIII recombinante	Hemobrás	Hemoderivado	2016	102.500.000	117.875.000,00	1,4712	173.417.700,00
Fator VIII recombinante	Hemobrás	Hemoderivado	2017	85.747.250	95.179.447,50	1,4083	134.041.215,91
Fator VIII recombinante	Hemobrás	Hemoderivado	2017	64.500.000	71.595.000,00	1,4083	100.827.238,50
Fator VIII recombinante	Hemobrás	Hemoderivado	2017	342.989.000	394.437.350,00	1,4083	555.486.120,01
Fator VIII recombinante	Hemobrás	Hemoderivado	2017	258.000.000	296.700.000,00	1,4083	417.842.610,00
Fator VIII recombinante	Hemobrás	Hemoderivado	2018	720.000.000	864.000.000,00	1,3788	1.191.283.200,00
Fator VIII recombinante	Hemobrás	Hemoderivado	2018	385.500.000	427.905.000,00	1,3788	589.995.414,00
Fator VIII recombinante	Hemobrás	Hemoderivado	2019	720.000.000	781.920.000,00	1,3413	1.048.789.296,00
Fator VIII recombinante	Hemobrás	Hemoderivado	2020	180.000.000	195.480.000,00	1,2982	253.772.136,00
Fator VIII recombinante	Hemobrás	Hemoderivado	2021	580.000.000	794.600.000,00	1,253	995.633.800,00
Fator VIII recombinante	Hemobrás	Hemoderivado	2022	500.000.000	550.000.000,00	1,1524	633.820.000,00
Fator VIII recombinante	Hemobrás	Hemoderivado	2023	670.000.000	663.300.000,00	1,0945	725.981.850,00
Fingolimode	NUPLAM	Sintético	2022	895.384	36.952.497,68	1,1524	42.584.058,33
Fingolimode	NUPLAM	Sintético	2022	966.924	37.903.420,80	1,1524	43.679.902,13
Fingolimode	NUPLAM	Sintético	2024	1.127.364	39.773.401,92	1,0483	41.694.457,23
Galantamina	FURP	Sintético	2018	1.070.440	4.549.370,00	1,3788	6.272.671,36
Galantamina	FURP	Sintético	2018	1.996.204	9.322.272,68	1,3788	12.853.549,57
Galantamina	FURP	Sintético	2018	1.045.716	4.716.179,16	1,3788	6.502.667,83
Galantamina	FURP	Sintético	2019	959.040	3.864.931,20	1,3413	5.184.032,22
Galantamina	FURP	Sintético	2019	2.566.200	10.110.828,00	1,3413	13.561.653,60
Galantamina	FURP	Sintético	2019	1.903.680	7.348.204,80	1,3413	9.856.147,10
Galantamina	FURP	Sintético	2021	1.325.772	5.077.706,76	1,253	6.362.366,57
Galantamina	FURP	Sintético	2021	1.920.744	7.183.582,56	1,253	9.001.028,95
Galantamina	FURP	Sintético	2021	1.131.956	4.154.278,52	1,253	5.205.310,99
Galantamina	FURP	Sintético	2022	1.222.088	4.485.062,96	1,1524	5.168.586,56
Galantamina	FURP	Sintético	2022	2.315.684	9.008.010,76	1,1524	10.380.831,60
Galantamina	FURP	Sintético	2022	1.526.840	5.175.987,60	1,1524	5.964.808,11
Galantamina	FURP	Sintético	2023	1.581.664	5.472.557,44	1,0945	5.989.714,12
Galantamina	FURP	Sintético	2023	2.122.736	7.854.123,20	1,0945	8.596.337,84
Galantamina	FURP	Sintético	2023	1.446.088	4.757.629,52	1,0945	5.207.225,51
Golimumabe	Fiocruz/Bio-Manguinhos	Biotecnológico	2020	104.478	119.387.011,00	1,2982	154.988.217,68
Golimumabe	Fiocruz/Bio-Manguinhos	Biotecnológico	2021	146.454	164.574.753,00	1,253	206.212.165,51
Golimumabe	Fiocruz/Bio-Manguinhos	Biotecnológico	2022	239.962	261.887.327,94	1,1524	301.798.956,72
Golimumabe	Fiocruz/Bio-Manguinhos	Biotecnológico	2023	263.050	283.394.392,22	1,0945	310.175.162,28
Golimumabe	Fiocruz/Bio-Manguinhos	Biotecnológico	2024	276.885	293.954.960,25	1,0483	308.152.984,83
Infliximabe	Fiocruz/Bio-Manguinhos	Biotecnológico	2014	178.969	164.715.908,84	1,642	270.463.522,32
Infliximabe	Fiocruz/Bio-Manguinhos	Biotecnológico	2015	44.743	41.179.667,48	1,5779	64.977.397,32
Infliximabe	Fiocruz/Bio-Manguinhos	Biotecnológico	2016	331.642	305.230.031,12	1,4712	449.054.421,78
Infliximabe	Fiocruz/Bio-Manguinhos	Biotecnológico	2016	358.331	323.196.645,45	1,4712	475.486.904,79
Infliximabe	Fiocruz/Bio-Manguinhos	Biotecnológico	2017	281.290	248.134.347,70	1,4083	349.447.601,87
Infliximabe	Fiocruz/Bio-Manguinhos	Biotecnológico	2018	383.038	324.371.899,00	1,3788	447.243.974,34
Infliximabe	Fiocruz/Bio-Manguinhos	Biotecnológico	2019	434.042	360.675.881,00	1,3413	483.774.559,19
Infliximabe	Fiocruz/Bio-Manguinhos	Biotecnológico	2020	239.748	193.140.989,00	1,2982	250.735.631,92
Infliximabe	Fiocruz/Bio-Manguinhos	Biotecnológico	2021	97.006	78.148.033,60	1,253	97.919.486,10
Infliximabe	Fiocruz/Bio-Manguinhos	Biotecnológico	2021	232.407	187.949.865,00	1,253	235.501.180,85
Infliximabe	Fiocruz/Bio-Manguinhos	Biotecnológico	2021	59.937	48.285.247,20	1,253	60.501.414,74
Infliximabe	Fiocruz/Bio-Manguinhos	Biotecnológico	2022	65.683	53.118.498,93	1,1524	61.213.758,17
Infliximabe	Fiocruz/Bio-Manguinhos	Biotecnológico	2023	126.596	102.379.451,16	1,0945	112.054.309,29
Infliximabe	Fiocruz/Bio-Manguinhos	Biotecnológico	2023	300.828	249.654.179,00	1,0945	273.246.498,92
Infliximabe	Fiocruz/Bio-Manguinhos	Biotecnológico	2024	365.836	299.180.680,80	1,0483	313.631.107,68
Insulina (NPH)	Bahiafarma	Biotecnológico	2018	16.253.674	169.444.551,60	1,3788	233.630.147,75
Insulina (regular)	Bahiafarma	Biotecnológico	2018	3.276.471	34.402.945,50	1,3788	47.434.781,26
Insulina (regular)	Bahiafarma	Biotecnológico	2019	819.117	8.477.860,95	1,3413	11.371.354,89
Insulina humana recombinante	Fiocruz/Farmanguinhos	Biotecnológico	2013	10.600.000	122.642.000,00	1,7011	208.626.306,20
Leflunomida	LFM	Sintético	2014	14.450.250	65.026.125,00	1,642	106.772.897,25
Leflunomida	LFM	Sintético	2016	19.304.160	78.567.931,20	1,4712	115.589.140,38
Leflunomida	LFM	Sintético	2018	16.485.960	63.606.130,00	1,3788	87.700.132,04
Leflunomida	LFM	Sintético	2019	20.470.980	75.032.283,00	1,3413	100.640.801,19
Mesilato de imatinibe	Fiocruz/Farmanguinhos	Sintético	2013	303.360	5.044.876,80	1,7011	8.581.839,92
Mesilato de imatinibe	Fiocruz/Farmanguinhos	Sintético	2013	1.568.340	104.357.343,60	1,7011	177.522.277,20
Mesilato de imatinibe	IVB	Sintético	2013	268.080	4.694.080,80	1,7011	7.985.100,85
Mesilato de imatinibe	IVB	Sintético	2013	1.680.000	117.667.200,00	1,7011	200.163.673,92
Mesilato de imatinibe	IVB	Sintético	2013	276.990	19.400.379,60	1,7011	33.001.985,74
Mesilato de imatinibe	Fiocruz/Farmanguinhos	Sintético	2014	639.060	10.090.757,40	1,642	16.569.023,65
Mesilato de imatinibe	Fiocruz/Farmanguinhos	Sintético	2014	1.815.420	114.752.698,20	1,642	188.423.930,44
Mesilato de imatinibe	IVB	Sintético	2014	303.360	5.044.876,80	1,642	8.283.687,71
Mesilato de imatinibe	IVB	Sintético	2014	639.060	10.090.757,40	1,642	16.569.023,65
Mesilato de imatinibe	IVB	Sintético	2014	1.276.740	84.954.279,60	1,642	139.494.927,10
Mesilato de imatinibe	IVB	Sintético	2014	1.815.420	114.752.698,20	1,642	188.423.930,44
Mesilato de imatinibe	Fiocruz/Farmanguinhos	Sintético	2016	453.480	5.895.240,00	1,4712	8.673.077,09
Mesilato de imatinibe	Fiocruz/Farmanguinhos	Sintético	2016	1.558.710	81.052.920,00	1,4712	119.245.055,90
Mesilato de imatinibe	IVB	Sintético	2016	453.480	5.895.240,00	1,4712	8.673.077,09
Mesilato de imatinibe	Fiocruz/Farmanguinhos	Sintético	2017	579.180	7.152.873,00	1,4083	10.073.391,05
Mesilato de imatinibe	Fiocruz/Farmanguinhos	Sintético	2017	2.646.660	130.745.004,00	1,4083	184.128.189,13
Mesilato de imatinibe	IVB	Sintético	2017	113.340	1.473.420,00	1,4083	2.075.017,39
Mesilato de imatinibe	IVB	Sintético	2017	1.558.710	81.052.920,00	1,4083	114.146.827,24
Mesilato de imatinibe	IVB	Sintético	2017	389.670	20.262.840,00	1,4083	28.536.157,57
Mesilato de imatinibe	IVB	Sintético	2018	193.020	2.383.797,00	1,3788	3.286.779,30
Mesilato de imatinibe	IVB	Sintético	2018	882.240	43.582.656,00	1,3788	60.091.766,09
Micofenolato de sódio	FURP	Sintético	2014	3.887.040	9.068.464,32	1,642	14.890.418,41
Micofenolato de sódio	FURP	Sintético	2014	41.352.840	198.721.072,62	1,642	326.300.001,24
Micofenolato de sódio	FURP	Sintético	2015	4.024.800	8.920.566,72	1,5779	14.075.762,23
Micofenolato de sódio	FURP	Sintético	2015	39.574.800	180.666.876,96	1,5779	285.074.265,16
Micofenolato de sódio	LQFEx	Sintético	2016	4.499.280	8.953.567,20	1,4712	13.172.488,06
Micofenolato de sódio	LQFEx	Sintético	2016	36.913.200	146.914.536,00	1,4712	216.140.665,36
Micofenolato de sódio	LQFEx	Sintético	2018	4.463.520	7.007.726,40	1,3788	9.662.253,16
Micofenolato de sódio	LQFEx	Sintético	2018	3.155.040	4.701.009,60	1,3788	6.481.752,04
Micofenolato de sódio	LQFEx	Sintético	2018	10.397.520	28.593.180,00	1,3788	39.424.276,58
Micofenolato de sódio	LQFEx	Sintético	2018	39.806.640	103.895.330,40	1,3788	143.250.881,56
Micofenolato de sódio	LQFEx	Sintético	2019	5.095.800	7.236.036,00	1,3413	9.705.695,09
Micofenolato de sódio	LQFEx	Sintético	2019	34.042.920	84.426.441,60	1,3413	113.241.186,12
Micofenolato de sódio	LQFEx	Sintético	2020	4.412.160	6.265.267,20	1,2982	8.133.569,88
Micofenolato de sódio	LQFEx	Sintético	2020	44.331.360	109.941.772,80	1,2982	142.726.409,45
Micofenolato de sódio	LQFEx	Sintético	2022	2.550.600	2.831.166,00	1,1524	3.262.635,70
Micofenolato de sódio	LQFEx	Sintético	2022	24.071.000	48.142.000,00	1,1524	55.478.840,80
Micofenolato de sódio	LQFEx	Sintético	2023	2.893.080	3.211.318,80	1,0945	3.514.788,43
Micofenolato de sódio	LQFEx	Sintético	2023	24.071.500	48.143.000,00	1,0945	52.692.513,50
Micronutrientes	LFM	Sintético	2014	20.000.000	2.500.000,00	1,642	4.105.000,00
Olanzapina	LAFEPE	Sintético	2012	17.826.816	163.650.170,88	1,7595	287.942.475,66
Olanzapina	LAFEPE	Sintético	2012	9.905.672	44.872.694,16	1,7595	78.953.505,37
Olanzapina	LAFEPE	Sintético	2014	15.940.120	138.710.924,24	1,642	227.763.337,60
Olanzapina	LAFEPE	Sintético	2014	18.313.512	78.638.220,53	1,642	129.123.958,11
Olanzapina	LAFEPE	Sintético	2015	31.991.318	97.573.519,90	1,5779	153.961.257,05
Olanzapina	LAFEPE	Sintético	2015	31.289.550	93.868.650,00	1,5779	148.115.342,84
Olanzapina	LAFEPE	Sintético	2015	19.149.046	37.532.130,16	1,5779	59.221.948,18
Olanzapina	LAFEPE	Sintético	2015	20.642.520	39.840.063,60	1,5779	62.863.636,35
Olanzapina	LAFEPE	Sintético	2016	30.815.460	87.824.061,00	1,4712	129.206.758,54
Olanzapina	LAFEPE	Sintético	2016	21.847.470	39.980.870,10	1,4712	58.819.856,09
Olanzapina	NUPLAM	Sintético	2016	13.206.620	37.638.867,00	1,4712	55.374.301,13
Olanzapina	NUPLAM	Sintético	2016	9.363.200	17.134.656,00	1,4712	25.208.505,91
Olanzapina	NUPLAM	Sintético	2018	21.447.150	54.261.289,50	1,3788	74.815.465,96
Olanzapina	NUPLAM	Sintético	2018	12.154.590	20.541.257,10	1,3788	28.322.285,29
Olanzapina	NUPLAM	Sintético	2019	19.231.200	48.078.000,00	1,3413	64.487.021,40
Olanzapina	NUPLAM	Sintético	2019	9.577.020	13.407.828,00	1,3413	17.983.919,70
Olanzapina	NUPLAM	Sintético	2020	22.020.870	46.023.618,50	1,2982	59.747.861,54
Olanzapina	NUPLAM	Sintético	2020	13.581.450	16.161.925,50	1,2982	20.981.411,68
Olanzapina	NUPLAM	Sintético	2021	13.370.010	26.539.469,85	1,253	33.253.955,72
Olanzapina	NUPLAM	Sintético	2021	6.098.850	6.891.700,50	1,253	8.635.300,73
Olanzapina	NUPLAM	Sintético	2023	15.715.110	27.972.895,80	1,0945	30.616.334,45
Olanzapina	NUPLAM	Sintético	2023	8.792.580	8.880.505,80	1,0945	9.719.713,60
Pramipexol	Fiocruz/Farmanguinhos	Sintético	2014	2.308.140	738.604,80	1,642	1.212.789,08
Pramipexol	Fiocruz/Farmanguinhos	Sintético	2014	18.121.290	13.047.328,80	1,642	21.423.713,89
Pramipexol	Fiocruz/Farmanguinhos	Sintético	2014	15.487.020	34.381.184,40	1,642	56.453.904,78
Pramipexol	Fiocruz/Farmanguinhos	Sintético	2015	2.676.450	813.640,80	1,5779	1.283.843,82
Pramipexol	Fiocruz/Farmanguinhos	Sintético	2015	20.484.120	14.011.138,08	1,5779	22.108.174,78
Pramipexol	Fiocruz/Farmanguinhos	Sintético	2015	18.145.440	38.268.732,96	1,5779	60.384.233,74
Pramipexol	Fiocruz/Farmanguinhos	Sintético	2016	2.794.710	768.545,25	1,4712	1.130.683,77
Pramipexol	Fiocruz/Farmanguinhos	Sintético	2016	15.962.640	9.848.948,88	1,4712	14.489.773,59
Pramipexol	Fiocruz/Farmanguinhos	Sintético	2016	15.801.300	30.022.470,00	1,4712	44.169.057,86
Pramipexol	Fiocruz/Farmanguinhos	Sintético	2017	1.302.690	340.002,09	1,4083	478.824,94
Pramipexol	Fiocruz/Farmanguinhos	Sintético	2017	17.892.420	10.484.958,12	1,4083	14.765.966,52
Pramipexol	Fiocruz/Farmanguinhos	Sintético	2017	17.340.810	31.300.162,05	1,4083	44.080.018,22
Pramipexol	Fiocruz/Farmanguinhos	Sintético	2020	2.528.000	435.068,80	1,2982	564.806,32
Pramipexol	Fiocruz/Farmanguinhos	Sintético	2020	16.592.000	7.962.500,80	1,2982	10.336.918,54
Pramipexol	Fiocruz/Farmanguinhos	Sintético	2020	18.062.500	29.774.225,00	1,2982	38.652.898,90
Quetiapina	LAFEPE	Sintético	2011	12.600.000	38.682.000,00	1,8245	70.575.309,00
Quetiapina	LAFEPE	Sintético	2011	16.300.000	89.813.000,00	1,8245	163.863.818,50
Quetiapina	LAFEPE	Sintético	2011	8.700.000	7.830.000,00	1,8245	14.285.835,00
Quetiapina	LAFEPE	Sintético	2012	18.862.788	55.022.752,60	1,7595	96.812.533,20
Quetiapina	LAFEPE	Sintético	2012	22.015.952	115.253.508,72	1,7595	202.788.548,59
Quetiapina	LAFEPE	Sintético	2012	13.995.968	11.966.552,64	1,7595	21.055.149,37
Quetiapina	LAFEPE	Sintético	2014	18.105.640	28.969.024,00	1,642	47.567.137,41
Quetiapina	LAFEPE	Sintético	2014	17.000.424	61.371.530,64	1,642	100.772.053,31
Quetiapina	LAFEPE	Sintético	2014	13.819.624	9.397.344,32	1,642	15.430.439,37
Quetiapina	LAFEPE	Sintético	2015	19.305.870	29.344.922,40	1,5779	46.303.353,05
Quetiapina	LAFEPE	Sintético	2015	18.292.980	62.744.921,40	1,5779	99.005.211,48
Quetiapina	LAFEPE	Sintético	2015	14.779.380	9.547.479,48	1,5779	15.064.967,87
Quetiapina	LAFEPE	Sintético	2016	21.897.300	29.955.506,40	1,4712	44.070.541,02
Quetiapina	LAFEPE	Sintético	2016	15.307.860	47.255.363,82	1,4712	69.522.091,25
Quetiapina	LAFEPE	Sintético	2016	18.140.700	10.547.002,98	1,4712	15.516.750,78
Rifampicina + isoniazida + pirazinamida + etambutol (4 em 1 tuberculostático)	Fiocruz/Farmanguinhos	Sintético	2015	24.000.480	9.157.863,15	1,5779	14.450.192,26
Rifampicina + isoniazida + pirazinamida + etambutol (4 em 1 tuberculostático)	Fiocruz/Farmanguinhos	Sintético	2018	12.060.000	3.955.680,00	1,3788	5.454.091,58
Rifampicina + isoniazida + pirazinamida + etambutol (4 em 1 tuberculostático)	Fiocruz/Farmanguinhos	Sintético	2019	26.073.720	12.869.988,00	1,3413	17.262.514,90
Rifampicina + isoniazida + pirazinamida + etambutol (4 em 1 tuberculostático)	Fiocruz/Farmanguinhos	Sintético	2020	30.888.000	18.146.700,00	1,2982	23.558.045,94
Rifampicina + isoniazida + pirazinamida + etambutol (4 em 1 tuberculostático)	Fiocruz/Farmanguinhos	Sintético	2022	35.824.320	22.211.078,40	1,1524	25.596.046,75
Riluzol	LFM	Sintético	2015	2.227.512	7.618.091,04	1,5779	12.020.585,85
Riluzol	LFM	Sintético	2017	889.000	2.951.480,00	1,4083	4.156.569,28
Riluzol	LFM	Sintético	2018	2.244.760	7.079.973,00	1,3788	9.761.866,77
Riluzol	LFM	Sintético	2019	2.600.136	7.790.787,00	1,3413	10.449.782,60
Riluzol	LFM	Sintético	2020	2.806.440	7.988.531,00	1,2982	10.370.710,94
Ritonavir termoestável	LAFEPE	Sintético	2017	55.127.520	54.576.244,80	1,4083	76.859.725,55
Ritonavir termoestável	LAFEPE	Sintético	2018	54.000.000	53.460.000,00	1,3788	73.710.648,00
Ritonavir termoestável	LAFEPE	Sintético	2018	50.001.000	49.500.990,00	1,3788	68.251.965,01
Ritonavir termoestável	LAFEPE	Sintético	2019	57.900.000	56.742.000,00	1,3413	76.108.044,60
Ritonavir termoestável	LAFEPE	Sintético	2020	80.040.000	78.439.200,00	1,2982	101.829.769,44
Ritonavir termoestável	LAFEPE	Sintético	2021	49.515.000	50.094.325,50	1,253	62.768.189,85
Ritonavir termoestável	LAFEPE	Sintético	2023	42.030.000	45.026.739,00	1,0945	49.281.765,84
Rituximabe	Fiocruz/Bio-Manguinhos	Biotecnológico	2020	37.186	12.069.460,02	1,2982	15.668.573,00
Rituximabe	Fiocruz/Bio-Manguinhos	Biotecnológico	2020	35.720	57.967.487,60	1,2982	75.253.392,40
Rituximabe	Fiocruz/Bio-Manguinhos	Biotecnológico	2021	29.456	9.385.859,80	1,253	11.760.482,33
Rituximabe	Fiocruz/Bio-Manguinhos	Biotecnológico	2021	30.080	48.004.371,20	1,253	60.149.477,11
Rituximabe	Fiocruz/Bio-Manguinhos	Biotecnológico	2022	23.292	7.181.855,28	1,1524	8.276.370,02
Rituximabe	Fiocruz/Bio-Manguinhos	Biotecnológico	2022	22.536	34.743.525,84	1,1524	40.038.439,18
Rituximabe	Fiocruz/Bio-Manguinhos	Biotecnológico	2023	23.998	35.977.086,46	1,0945	39.376.921,13
Rituximabe	Fiocruz/Bio-Manguinhos	Biotecnológico	2024	27.971	41.322.397,43	1,0483	43.318.269,23
Rivastigmina	IVB	Sintético	2012	6.325.500	13.220.295,00	1,7595	23.261.109,05
Rivastigmina	IVB	Sintético	2012	8.663.790	20.706.458,10	1,7595	36.433.013,03
Rivastigmina	IVB	Sintético	2012	4.431.240	12.097.285,20	1,7595	21.285.173,31
Rivastigmina	IVB	Sintético	2012	7.145.820	19.936.837,80	1,7595	35.078.866,11
Rivastigmina	IVB	Sintético	2014	7.303.740	14.534.442,60	1,642	23.865.554,75
Rivastigmina	IVB	Sintético	2014	10.364.910	23.528.345,70	1,642	38.633.543,64
Rivastigmina	IVB	Sintético	2014	5.299.590	13.725.938,10	1,642	22.537.990,36
Rivastigmina	IVB	Sintético	2014	8.757.360	23.207.004,00	1,642	38.105.900,57
Rivastigmina	IVB	Sintético	2015	3.862.860	7.300.805,40	1,5779	11.519.940,84
Rivastigmina	IVB	Sintético	2015	5.914.740	12.716.691,00	1,5779	20.065.666,73
Rivastigmina	IVB	Sintético	2015	3.039.480	7.477.120,80	1,5779	11.798.148,91
Rivastigmina	IVB	Sintético	2015	7.902.960	19.836.429,60	1,5779	31.299.902,27
Rivastigmina	IVB	Sintético	2016	3.647.820	6.566.076,00	1,4712	9.660.011,01
Rivastigmina	IVB	Sintético	2016	6.750.390	13.770.795,60	1,4712	20.259.594,49
Rivastigmina	IVB	Sintético	2016	3.231.030	7.560.610,20	1,4712	11.123.169,73
Rivastigmina	IVB	Sintético	2016	5.665.950	13.484.961,00	1,4712	19.839.074,62
Sevelâmer	Bahiafarma	Sintético	2015	68.930.100	77.201.712,00	1,5779	121.816.581,36
Sevelâmer	Bahiafarma	Sintético	2016	37.813.320	41.216.518,80	1,4712	60.637.742,46
Sevelâmer	Fiocruz/Farmanguinhos	Sintético	2016	37.813.320	41.216.519,00	1,4712	60.637.742,75
Sevelâmer	Bahiafarma	Sintético	2017	38.858.040	40.023.781,20	1,4083	56.365.491,06
Sevelâmer	Fiocruz/Farmanguinhos	Sintético	2017	38.858.040	40.023.781,00	1,4083	56.365.490,78
Sevelâmer	Fiocruz/Farmanguinhos	Sintético	2020	199.362.780	205.343.663,00	1,2982	266.577.143,31
Sildenafila	LFM	Sintético	2019	419.232	586.924,80	1,3413	787.242,23
Sildenafila	LFM	Sintético	2019	160.320	206.812,80	1,3413	277.398,01
Sildenafila	LFM	Sintético	2019	5.772.266	20.823.502,00	1,3413	27.930.563,23
Sildenafila	LFM	Sintético	2021	6.189.240	20.362.599,60	1,253	25.514.337,30
Sildenafila	LFM	Sintético	2022	1.547.280	4.842.986,40	1,1524	5.581.057,53
Sildenafila	LFM	Sintético	2022	5.703.060	17.850.577,80	1,1524	20.571.005,86
Sofosbuvir	Fiocruz/Farmanguinhos	Sintético	2023	400.400	11.811.800,00	1,0945	12.928.015,10
Sofosbuvir	Fiocruz/Farmanguinhos	Sintético	2024	899.612	26.538.554,00	1,0483	27.820.366,16
Sofosbuvir	FURP	Sintético	2024	324.996	9.012.139,08	1,0483	9.447.425,40
Somatropina	Fiocruz/Bio-Manguinhos	Biotecnológico	2020	16.922.880	86.983.603,00	1,2982	112.922.113,41
Somatropina	Fiocruz/Bio-Manguinhos	Biotecnológico	2020	16.922.900	86.983.706,00	1,2982	112.922.247,13
Somatropina	Fiocruz/Bio-Manguinhos	Biotecnológico	2021	18.564.420	93.806.014,00	1,253	117.538.935,54
Somatropina	Fiocruz/Bio-Manguinhos	Biotecnológico	2021	20.821.500	105.211.039,50	1,253	131.829.432,49
Somatropina	Fiocruz/Bio-Manguinhos	Biotecnológico	2021	5.962.840	30.130.230,52	1,253	37.753.178,84
Somatropina	Fiocruz/Bio-Manguinhos	Biotecnológico	2023	28.610.700	148.355.062,71	1,0945	162.374.616,14
Somatropina	Fiocruz/Bio-Manguinhos	Biotecnológico	2023	12.753.180	66.129.064,25	1,0945	72.378.260,82
Somatropina	Fiocruz/Bio-Manguinhos	Biotecnológico	2024	25.864.824	132.169.250,64	1,0483	138.553.025,45
Somatropina	Fiocruz/Bio-Manguinhos	Biotecnológico	2024	11.041.112	56.420.082,32	1,0483	59.145.172,30
Somatropina	Fiocruz/Bio-Manguinhos	Biotecnológico		0			
Tacrolimo	Fiocruz/Farmanguinhos	Sintético	2011	28.980.000	86.940.000,00	1,8245	158.622.030,00
Tacrolimo	Fiocruz/Farmanguinhos	Sintético	2011	1.020.000	14.851.200,00	1,8245	27.096.014,40
Tacrolimo	Fiocruz/Farmanguinhos	Sintético	2012	53.547.100	152.609.235,00	1,7595	268.515.948,98
Tacrolimo	Fiocruz/Farmanguinhos	Sintético	2012	2.965.050	41.006.641,50	1,7595	72.151.185,72
Tacrolimo	Fiocruz/Farmanguinhos	Sintético	2013	55.885.200	151.448.892,00	1,7011	257.629.710,18
Tacrolimo	Fiocruz/Farmanguinhos	Sintético	2013	2.540.100	33.376.914,00	1,7011	56.777.468,41
Tacrolimo	Fiocruz/Farmanguinhos	Sintético	2015	55.357.300	126.214.644,00	1,5779	199.154.086,77
Tacrolimo	Fiocruz/Farmanguinhos	Sintético	2015	3.259.650	36.410.290,50	1,5779	57.451.797,38
Tacrolimo	Fiocruz/Farmanguinhos	Sintético	2016	64.119.400	139.139.098,00	1,4712	204.701.440,98
Tacrolimo	Fiocruz/Farmanguinhos	Sintético	2016	4.532.400	48.088.764,00	1,4712	70.748.189,60
Tenofovir	FUNED	Sintético	2011	28.800.000	115.776.000,00	1,8245	211.233.312,00
Tenofovir	LAFEPE	Sintético	2011	7.200.000	28.944.000,00	1,8245	52.808.328,00
Tenofovir	FUNED	Sintético	2012	19.800.000	79.596.000,00	1,7595	140.049.162,00
Tenofovir	LAFEPE	Sintético	2012	19.800.000	79.596.000,00	1,7595	140.049.162,00
Tenofovir	FUNED	Sintético	2013	16.500.000	63.013.500,00	1,7011	107.192.264,85
Tenofovir	LAFEPE	Sintético	2013	16.500.000	63.013.500,00	1,7011	107.192.264,85
Tenofovir	FUNED	Sintético	2014	24.000.000	87.072.000,00	1,642	142.972.224,00
Tenofovir	LAFEPE	Sintético	2014	24.000.000	87.072.000,00	1,642	142.972.224,00
Tenofovir	FUNED	Sintético	2015	9.990.000	34.431.534,00	1,5779	54.329.517,50
Tenofovir	LAFEPE	Sintético	2015	16.650.000	57.385.890,00	1,5779	90.549.195,83
Tenofovir + lamivudina (2 em 1)	Fiocruz/Farmanguinhos	Sintético	2014	43.000.000	129.000.000,00	1,642	211.818.000,00
Tenofovir + lamivudina (2 em 1)	Fiocruz/Farmanguinhos	Sintético	2015	54.439.860	163.319.580,00	1,5779	257.701.965,28
Tenofovir + lamivudina (2 em 1)	Fiocruz/Farmanguinhos	Sintético	2016	56.581.710	147.112.446,00	1,4712	216.431.830,56
Tenofovir + lamivudina (2 em 1)	Fiocruz/Farmanguinhos	Sintético	2017	159.999.990	303.999.981,00	1,4083	428.123.173,24
Tenofovir + lamivudina (2 em 1)	Fiocruz/Farmanguinhos	Sintético	2019	54.000.000	118.260.000,00	1,3413	158.622.138,00
Tenofovir + lamivudina (2 em 1)	LAFEPE	Sintético	2021	67.500.000	147.825.000,00	1,253	185.224.725,00
Tenofovir + lamivudina (2 em 1)	LAFEPE	Sintético	2022	78.000.000	170.820.000,00	1,1524	196.852.968,00
Tenofovir + lamivudina (2 em 1)	LAFEPE	Sintético	2023	19.500.000	42.705.000,00	1,0945	46.740.622,50
Tenofovir + lamivudina (2 em 1)	LAFEPE	Sintético	2023	43.000.020	96.320.044,80	1,0945	105.422.289,03
Tenofovir + lamivudina (2 em 1)	LAFEPE	Sintético	2023	10.749.990	24.079.977,60	1,0945	26.355.535,48
Tenofovir + lamivudina (2 em 1)	LAFEPE	Sintético	2024	82.003.500	188.608.050,00	1,0483	197.717.818,82
Tenofovir + lamivudina (2 em 1)	LAFEPE	Sintético	2024	20.500.860	47.151.978,00	1,0483	49.429.418,54
Toxina botulínica	LAFEPE	Biotecnológico	2014	36.276	14.954.781,00	1,642	24.555.750,40
Trastuzumabe	TECPAR	Biotecnológico	2018	172.374	222.896.819,40	1,3788	307.330.134,59
Trastuzumabe	TECPAR	Biotecnológico	2018	161.668	151.796.551,92	1,3788	209.297.085,79
Trastuzumabe	Fiocruz/Bio-Manguinhos	Biotecnológico	2020	198.522	177.546.165,00	1,2982	230.490.431,40
Trastuzumabe	Fiocruz/Bio-Manguinhos	Biotecnológico	2021	226.200	203.998.470,00	1,253	255.610.082,91
Trastuzumabe	Fiocruz/Bio-Manguinhos	Biotecnológico	2022	235.953	218.464.163,64	1,1524	251.758.102,18
Trastuzumabe	Fiocruz/Bio-Manguinhos	Biotecnológico	2023	265.056	237.802.332,45	1,0945	260.274.652,87
Trastuzumabe	Fiocruz/Bio-Manguinhos	Biotecnológico	2024	580.000	512.783.800,00	1,0483	537.551.257,54
Vacina adsorvida contra hepatite A (inativada)	Instituto Butantan	Vacina	2014	5.600.000	111.160.000,00	1,642	182.524.720,00
Vacina adsorvida contra hepatite A (inativada)	Instituto Butantan	Vacina	2016	4.500.000	157.140.000,00	1,4712	231.184.368,00
Vacina adsorvida contra hepatite A (inativada)	Instituto Butantan	Vacina	2017	4.500.000	142.740.000,00	1,4083	201.020.742,00
Vacina adsorvida contra hepatite A (inativada)	Instituto Butantan	Vacina	2018	3.000.000	99.000.000,00	1,3788	136.501.200,00
Vacina adsorvida contra hepatite A (inativada)	Instituto Butantan	Vacina	2020	4.500.000	156.645.000,00	1,2982	203.356.539,00
Vacina adsorvida contra hepatite A (inativada)	Instituto Butantan	Vacina	2021	3.500.000	146.195.000,00	1,253	183.182.335,00
Vacina DTPa (vacina adsorvida contra difteria, tétano e pertussis acelular)	Instituto Butantan	Vacina	2016	5.000.000	218.500.000,00	1,4712	321.457.200,00
Vacina DTPa (vacina adsorvida contra difteria, tétano e pertussis acelular)	Instituto Butantan	Vacina	2017	5.000.000	196.650.000,00	1,4083	276.942.195,00
Vacina DTPa (vacina adsorvida contra difteria, tétano e pertussis acelular)	Instituto Butantan	Vacina	2018	2.000.000	76.280.000,00	1,3788	105.174.864,00
Vacina DTPa (vacina adsorvida contra difteria, tétano e pertussis acelular)	Instituto Butantan	Vacina	2019	4.000.000	172.240.000,00	1,3413	231.025.512,00
Vacina DTPa (vacina adsorvida contra difteria, tétano e pertussis acelular)	Instituto Butantan	Vacina	2021	4.000.000	206.680.000,00	1,253	258.970.040,00
Vacina HPV	Instituto Butantan	Vacina	2014	15.000.000	465.300.000,00	1,642	764.022.600,00
Vacina HPV	Instituto Butantan	Vacina	2015	11.000.000	465.355.000,00	1,5779	734.283.654,50
Vacina HPV	Instituto Butantan	Vacina	2016	6.000.000	288.420.000,00	1,4712	424.323.504,00
Vacina HPV	Instituto Butantan	Vacina	2017	6.000.000	258.120.000,00	1,4083	363.510.396,00
Vacina HPV	Instituto Butantan	Vacina	2018	14.000.000	567.420.000,00	1,3788	782.358.696,00
Vacina HPV	Instituto Butantan	Vacina	2019	6.500.000	252.915.000,00	1,3413	339.234.889,50
Vacina Influenza	Instituto Butantan	Vacina	2011	32.750.000	229.250.000,00	1,8245	418.266.625,00
Vacina Influenza	Instituto Butantan	Vacina	2012	33.900.000	260.352.000,00	1,7595	458.089.344,00
Vacina Influenza	Instituto Butantan	Vacina	2013	44.000.000	382.360.000,00	1,7011	650.432.596,00
Vacina Influenza	Instituto Butantan	Vacina	2014	54.000.000	455.760.000,00	1,642	748.357.920,00
Vacina tetraviral (MMRV)	Fiocruz/Bio-Manguinhos	Vacina	2015	1.560.990	57.569.311,20	1,5779	90.838.616,14
Vacina tetraviral (MMRV)	Fiocruz/Bio-Manguinhos	Vacina	2015	901.730	41.274.887,29	1,5779	65.127.644,65
Vacina tetraviral (MMRV)	Fiocruz/Bio-Manguinhos	Vacina	2016	1.998.270	97.735.385,70	1,4712	143.788.299,44
Vacina tetraviral (MMRV)	Fiocruz/Bio-Manguinhos	Vacina	2017	1.917.730	93.796.174,30	1,4083	132.093.152,27
Ziprasidona	LFM	Sintético	2017	2.505.540	7.266.066,00	1,4083	10.232.800,75
Ziprasidona	LFM	Sintético	2017	4.632.840	19.828.555,20	1,4083	27.924.554,29
Ziprasidona	LFM	Sintético	2018	2.142.000	5.783.400,00	1,3788	7.974.151,92
Ziprasidona	LFM	Sintético	2018	3.018.780	11.924.181,00	1,3788	16.441.060,76
Ziprasidona	LFM	Sintético	2019	1.984.350	5.179.153,50	1,3413	6.946.798,59
Ziprasidona	LFM	Sintético	2019	3.277.380	12.683.460,50	1,3413	17.012.325,57
Ziprasidona	LFM	Sintético	2020	2.423.610	6.010.552,80	1,2982	7.802.899,64
Ziprasidona	LFM	Sintético	2020	3.544.560	13.043.980,80	1,2982	16.933.695,87
Ziprasidona	LFM	Sintético	2021	2.485.110	5.864.859,60	1,253	7.348.669,08
Ziprasidona	LFM	Sintético	2021	3.666.960	12.797.690,40	1,253	16.035.506,07
Ziprasidona	LFM	Sintético	2022	1.835.232	4.110.919,60	1,1524	4.737.423,75
Ziprasidona	LFM	Sintético	2022	3.029.849	10.059.098,00	1,1524	11.592.104,54

Bahiafarma: Fundação Baiana de Pesquisa Científica e Desenvolvimento Tecnológico, Fornecimento e Distribuição de Medicamentos; Bio-Manguinhos: Instituto de Tecnologia em Imunobiológicos; Farmanguinhos: Instituto de Tecnologia de Fármacos; Fiocruz: Fundação Oswaldo Cruz; FUNED: Fundação Ezequiel Dias; FURP: Fundação para o Remédio Popular; Hemobrás: Empresa Brasileira de Hemoderivados e Biotecnologia; IVB: Instituto Vital Brazil; LAFEPE: Laboratório Farmacêutico de Pernambuco; LFM: Laboratório Farmacêutico da Marinha; LQFEx: Laboratório Químico-Farmacêutico do Exército; NUPLAM: Núcleo de Pesquisa em Alimentos e Medicamentos da Universidade Federal do Rio Grande do Norte.

Fonte: elaboração própria com base no Ministério da Saúde (2025).

Verificou-se que, em algumas parcerias, particularmente aquelas relacionadas a medicamentos sintéticos com menor complexidade produtiva, houve estabilidade nas compras públicas, com fornecimento regular por parte dos laboratórios públicos e redução progressiva da participação de fornecedores privados. Em contraste, outras PDPs apresentaram forte volatilidade nas aquisições. Casos ilustrativos são os do tenofovir e da clozapina, nos quais observou-se alternância entre compras do parceiro público e aquisições junto a fornecedores privados, inclusive por meio de pregão, dispensa ou inexigibilidade de licitação, mesmo em períodos de vigência das parcerias. No caso do tenofovir, por exemplo, identificaram-se períodos prolongados sem aquisições dos parceiros públicos (2016-2021), concomitantes à realização de múltiplos contratos com empresas privadas, evidenciando descontinuidade no padrão de compras públicas.

Em determinados medicamentos, o Ministério da Saúde alternou compras do parceiro público com aquisições por dispensa ou inexigibilidade de licitação junto a empresas privadas, inclusive em períodos nos quais a PDP estava formalmente ativa. Essas observações indicam que a existência de uma PDP não garantiu, por si só, a migração das aquisições junto a fornecedores privados por licitações regulares para compras da produção pública.

Foram identificados ainda casos em que as aquisições do parceiro público cessaram por longos intervalos, sem que houvesse retomada consistente da produção nacional. Em alguns desses casos, a interrupção das compras públicas coincidiu com a suspensão ou extinção da PDP; em outros, ocorreu mesmo com a parceria formalmente vigente, sugerindo problemas de capacidade produtiva, dificuldades operacionais ou mudanças na estratégia de compras governamentais.

### Sustentabilidade produtiva e tecnológica das PDPs

O Quadro 3 sintetiza os resultados obtidos por meio dos indicadores de sustentabilidade produtiva e tecnológica construídos neste estudo, elencando o indicador e o achado principal correspondente.

Os indicadores sintetizados no Quadro 3 permitem uma leitura integrada do desempenho produtivo e tecnológico das PDPs entre 2011 e 2024, evidenciando avanços relevantes, mas também entraves estruturais persistentes. Do ponto de vista produtivo, observa-se uma consolidação incompleta da capacidade nacional, uma vez que apenas 46,3% das PDPs em vigor alcançaram a fase IV, correspondente à efetiva internalização produtiva. O tempo médio de transição da fase III para a fase IV varia entre cinco anos para medicamentos sintéticos e sete para biológicos, indicando um processo de maturação lento, desigual e condicionado pela complexidade tecnológica e pelas capacidades institucionais dos LFOs.

**Quadro 3:** Indicadores de sustentabilidade produtiva e tecnológica das Parcerias para o Desenvolvimento Produtivo (PDPs).

DIMENSÃO PRODUTIVA	
INDICADOR	ACHADO PRINCIPAL
(1) Percentual de PDPs em fase IV (internalização produtiva)	36 em fase IV de 82 PDPs vigentes e suspensas → ≈ 46,34%
(2) Tempo médio de transição da fase III → fase IV	Variação entre 5-7 anos
(3) Taxa de extinção e suspensão de PDPs	((66+7) / 152) *100 = 48% dos projetos encerrados antes da conclusão
(4) Percentual de PDPs por plataforma tecnológica	Sintéticos (73,9%), biotecnológicos (19,3%), vacinas (5,7%), hemoderivados (1,1%)
(5) Participação relativa de LFOs na fase IV	Bio-Manguinhos (30%), Farmanguinhos (25%), Instituto Butantan (15%), Hemobrás (10%), demais (20%)
DIMENSÃO TECNOLÓGICA	
INDICADOR	DESCRIÇÃO/ACHADO PRINCIPAL
(6) Índice de complexidade tecnológica	(20/82) *100 = 24,3%
(7) Tempo médio de maturação tecnológica por plataforma	Sintéticos: 5 anos, biotecnológicos: 7 anos, vacinas: 6 anos
(8) Taxa de sucesso tecnológico (fase III → fase IV)	38/56 = 68%
(9) Percentual de produtos internalizados com produção contínua	(17/38) * 100 = 44,7%

Bio-Manguinhos: Instituto de Tecnologia em Imunobiológicos; Farmanguinhos: Instituto de Tecnologia de Fármacos; Hemobrás: Empresa Brasileira de Hemoderivados e Biotecnologia; LFOs: laboratórios farmacêuticos oficiais.

Fonte: elaboração própria.

A elevada taxa de extinção e suspensão das parcerias, que atinge 48%, reforça a fragilidade institucional da política. A análise por plataforma tecnológica revela forte concentração em medicamentos sintéticos (73,9%) e os biotecnológicos representam 19,3%, vacinas 5,7% e hemoderivados apenas 1,1%, evidenciando baixa inserção em tecnologias de maior fronteira. Na fase IV, a produção internalizada está concentrada em poucos LFOs: Bio-Manguinhos responde por cerca de 30% das PDPs maduras, seguido por Farmanguinhos (25%), Instituto Butantan (15%) e Hemobrás (10%), e os demais laboratórios somam apenas 20%, refletindo desigualdades estruturais de infraestrutura produtiva, capacidade regulatória e domínio tecnológico.

No âmbito tecnológico, o índice de complexidade de 24,3% indica que pouco mais de um quarto das PDPs envolve produtos de elevada densidade tecnológica. Os tempos médios de maturação variam entre as plataformas - cerca de cinco anos para sintéticos, sete para biotecnológicos e seis para vacinas -, e a taxa de sucesso tecnológico alcança 68% entre as parcerias que avançaram da fase III para a internalização. Além disso, 46,3% dos produtos internalizados apresentam produção contínua, sinalizando sustentabilidade nas PDPs bem-sucedidas. Em conjunto, os resultados mostram que as PDPs contribuíram para a expansão da produção pública e para o abastecimento estratégico do SUS, mas permanecem limitadas por concentração em poucos laboratórios públicos da rede oficial, baixa taxa de inovação e restrita diversificação tecnológica, condicionando a sustentabilidade de longo prazo da política no âmbito do CEIS.

## Discussão

Os resultados apresentados evidenciam que as PDPs constituíram um instrumento relevante da política industrial em saúde no Brasil, produzindo avanços concretos na internalização produtiva de determinados medicamentos estratégicos para o SUS. Contudo, os achados também indicam que tais avanços ocorreram de forma heterogênea, seletiva e estruturalmente limitada, refletindo condicionantes institucionais, tecnológicos e macroestruturais que extrapolam o desenho formal da política.

Um primeiro elemento central da discussão refere-se à assimetria institucional observada na execução das PDPs. A elevada concentração das parcerias, dos valores contratados e das aquisições governamentais em um número restrito de LFOs revela que a política não foi capaz de promover uma difusão ampla e equilibrada das capacidades produtivas ao longo da rede pública. Esse resultado confirma achados prévios da literatura, segundo os quais a existência de laboratórios públicos não implica, automaticamente, capacidade homogênea de absorção tecnológica e execução de políticas industriais complexas ^9^ ^,^ ^15^.

Ademais, essa heterogeneidade institucional está associada não apenas a diferenças de capacidade produtiva instalada, mas também às distintas naturezas jurídicas, arranjos de governança e níveis de dependência em relação a políticas estaduais ou federais. No caso brasileiro, os LFOs apresentam grande diversidade organizacional, incluindo autarquias, fundações públicas, unidades da administração direta e sociedades de economia mista, o que implica diferentes graus de autonomia administrativa, flexibilidade gerencial e capacidade de resposta às demandas do Setor Saúde ^16^. Tais fatores influenciam diretamente a capacidade dos laboratórios públicos de realizar investimentos contínuos, atender a requisitos regulatórios crescentes e sustentar processos de internalização tecnológica, especialmente em segmentos de maior complexidade. Além disso, diagnósticos sobre o setor apontam limitações recorrentes relacionadas à rigidez administrativa, dificuldades na aquisição de insumos, restrições na contratação e qualificação de recursos humanos, e baixa capacidade de investimento em pesquisa e desenvolvimento, elementos que comprometem a capacidade de adaptação e inovação desses laboratórios ^16^. Nesse sentido, as PDPs, embora relevantes, não representam, isoladamente, um instrumento suficiente para a capacitação estrutural dos laboratórios públicos regionais.

Essa concentração institucional sugere que as PDPs tenderam a reforçar as capacidades previamente existentes, em vez de promover um processo sistemático de fortalecimento estrutural do conjunto dos laboratórios públicos. Embora esse padrão possa ser interpretado, em parte, como uma estratégia pragmática de reduzir riscos e garantir o fornecimento ao SUS, ele limita o potencial sistêmico da política e sua capacidade de induzir desenvolvimento regional, diversificação produtiva e aprendizado tecnológico disseminado.

Outro achado relevante diz respeito à instabilidade na execução das parcerias, expressa pela permanência prolongada na fase III, pela ocorrência de suspensões e pela irregularidade das aquisições governamentais. Esses resultados indicam que a trajetória das PDPs esteve longe de seguir um percurso linear de maturação tecnológica, sendo marcada por descontinuidades que comprometeram processos cumulativos de aprendizagem. Tal instabilidade reforça a compreensão de que políticas industriais em saúde exigem horizontes temporais longos, estabilidade regulatória e coordenação interinstitucional consistente, sob pena de interromper investimentos e enfraquecer capacidades em formação ^1^.

Um elemento adicional relevante para a interpretação dos resultados refere-se ao contexto institucional e conjuntural no período analisado. Entre 2017 e 2022, observou-se uma inflexão na política de aquisições do Ministério da Saúde, com maior ênfase em critérios de economicidade de curto prazo e priorização de compras pelo menor preço, em detrimento de estratégias orientadas ao desenvolvimento produtivo ^17^. Essa mudança implicou, em diversos casos, a aquisição de medicamentos junto a fornecedores privados, mesmo na vigência de PDPs, fenômeno já identificado na literatura como expressão de fragilidades na governança e na coordenação da política ^8^ ^,^ ^9^. Tal dinâmica reduziu a previsibilidade da demanda para os laboratórios públicos e comprometeu a continuidade produtiva das PDPs. Considerando que esse período corresponde a aproximadamente metade da série histórica analisada, seus efeitos tendem a impactar de forma significativa os resultados observados no estudo.

Adicionalmente, a pandemia de COVID-19 introduziu um fator de perturbação relevante nas cadeias globais de suprimento e nas dinâmicas de produção e aquisição de medicamentos. A literatura tem demonstrado que o período foi marcado por disrupções logísticas, aumento da competição internacional por insumos estratégicos e reorientação das prioridades de compras públicas em saúde ^3^. Esse contexto reforçou a centralidade do debate sobre autonomia produtiva, ao mesmo tempo em que impôs restrições operacionais e institucionais que podem ter limitado o desempenho das PDPs em determinados momentos do período analisado.

A análise dos indicadores de sustentabilidade produtiva e tecnológica revela que a formalização de uma PDP não garantiu, por si só, autonomia produtiva efetiva. Mesmo em parcerias classificadas como concluídas ou em fase IV, observou-se a manutenção de aquisições junto a fornecedores privados, nacionais ou internacionais, sugerindo que a internalização tecnológica ocorreu, em muitos casos, de forma parcial. Esse achado aponta para limites do modelo de transferência de tecnologia adotado, especialmente quando não acompanhado por políticas complementares voltadas ao fortalecimento da base farmoquímica e ao domínio de etapas críticas do processo produtivo ^2^.

A distinção entre medicamentos sintéticos e biológicos mostrou ser particularmente elucidativa. Os resultados indicam que as PDPs associadas a medicamentos sintéticos apresentaram desempenho mais consistente em termos de estabilidade produtiva. Esse padrão é compatível com a literatura que destaca a menor complexidade tecnológica relativa dessas rotas produtivas e a maior experiência acumulada dos laboratórios públicos nesse segmento ^6^.

Em contraste, os resultados observados nas plataformas biotecnológicas indicam limites estruturais mais complexos e heterogêneos. Ainda que experiências como as parcerias envolvendo Bio-Manguinhos e a Bionovis (Companhia Brasileira de Biotecnologia Farmacêutica) representem avanços relevantes na construção de capacidades nacionais, os resultados indicam que, no período analisado, tais iniciativas não foram suficientes para produzir uma transformação estrutural ampla no segmento biotecnológico. Nesse sentido, os achados corroboram análises que apontam a necessidade de políticas integradas de ciência, tecnologia e inovação, capazes de articular transferência tecnológica, pesquisa local e desenvolvimento de fornecedores estratégicos, para além do instrumento das PDPs isoladamente ^8^ ^,^ ^10^.

Outro aspecto relevante diz respeito à distinção entre a internalização de etapas finais da produção de medicamentos e a efetiva autonomia tecnológica ao longo da cadeia produtiva. Os resultados sugerem que, embora as PDPs tenham sido capazes de ampliar a produção nacional de medicamentos acabados, sua contribuição para o fortalecimento da base farmoquímica - especialmente no que se refere à produção de IFAs - permaneceu limitada. Esse padrão reforça a interpretação de que a internalização produtiva observada nas PDPs ocorreu, em grande medida, nas etapas finais da cadeia, sem necessariamente implicar domínio tecnológico sobre componentes críticos do processo produtivo. Tal limitação é amplamente reconhecida na literatura sobre o CEIS, que aponta a dependência externa de IFAs como um dos principais gargalos estruturais para a consolidação da autonomia sanitária nacional ^1^ ^,^ ^2^.

Nesse sentido, cabe mencionar que a pandemia de COVID-19 evidenciou de forma contundente a vulnerabilidade das cadeias globais de suprimento em saúde e reforçou a centralidade da capacidade produtiva nacional como elemento estratégico para a sustentabilidade dos sistemas públicos ^3^. No caso brasileiro, esse contexto também expôs a dependência estrutural de insumos farmacêuticos ativos, historicamente associada à fragilidade da base farmoquímica nacional ^2^. Paralelamente, a literatura sobre políticas de inovação pelo lado da demanda destaca que o poder de compra do Estado constitui instrumento fundamental para a indução de capacidades produtivas e tecnológicas, desde que orientado por objetivos de desenvolvimento de longo prazo ^16^. No entanto, quando subordinado predominantemente a critérios de menor preço, esse instrumento pode gerar efeitos contraditórios, comprometendo a sustentabilidade de políticas industriais em saúde.

Outro aspecto central diz respeito à relação entre as PDPs e a política de compras governamentais. Os dados analisados neste estudo indicam que, em diversos casos, o Ministério da Saúde manteve aquisições junto a fornecedores privados e importações relevantes mesmo na vigência de PDPs e após o avanço de algumas parcerias para fases mais maduras. Essa coexistência é evidenciada pela persistência de volumes expressivos de compras externas nos períodos posteriores à implantação das PDPs, conforme observado nos dados de aquisições governamentais examinados. Esse comportamento evidencia uma tensão estrutural entre os objetivos de política industrial associados às PDPs - voltados à internalização produtiva e ao fortalecimento dos laboratórios públicos - e a lógica predominante das políticas de aquisição pública orientadas pela busca do menor preço no curto prazo. Em contextos de restrição fiscal ou de mudança de orientação governamental, a priorização de compras mais baratas junto a fornecedores privados tende a prevalecer, mesmo que isso implique a descontinuidade de processos de aprendizagem tecnológica e a fragilização da capacidade produtiva nacional. Tal dinâmica é particularmente relevante no período entre 2017 e 2022, no qual observa-se maior incidência desse padrão, contribuindo para a instabilidade das PDPs e para a limitação de seus efeitos estruturais.

Resultados semelhantes já foram apontados por estudos anteriores, que destacam limitações operacionais, produtivas e institucionais dos parceiros públicos para atender integralmente à demanda, especialmente em contextos de incerteza produtiva ou tecnológica ^8^ ^,^ ^9^ ^,^ ^10^. A manutenção prolongada dessa coexistência entre produção pública e aquisições externas tende a limitar os ganhos esperados em termos de autonomia sanitária e de racionalização do gasto público.

Do ponto de vista da avaliação de políticas públicas, os achados reforçam a pertinência da abordagem formativa adotada neste estudo. Ao analisar resultados intermediários e estruturais ao longo de um horizonte temporal ampliado, foi possível identificar gargalos, descontinuidades e padrões de desempenho que avaliações pontuais ou exclusivamente normativas tenderiam a obscurecer ^11^ ^,^ ^12^. Essa abordagem permite compreender as PDPs não apenas como instrumentos normativos, mas como políticas dinâmicas, sujeitas a ajustes, aprendizados e retrocessos.

Os resultados também dialogam com o debate mais amplo sobre o CEIS. Ao evidenciar que as PDPs produziram avanços relevantes, porém insuficientes para transformar estruturalmente a capacidade tecnológica do CEIS, o estudo reforça a compreensão de que políticas industriais em saúde precisam ser concebidas como políticas de Estado, articuladas a estratégias de longo prazo e protegidas de oscilações conjunturais ^3^ ^,^ ^13^. A instabilidade institucional observada ao longo do período analisado comprometeu processos de maturação tecnológica e fragilizou a sustentabilidade das parcerias, evidenciando a sensibilidade da política a mudanças de orientação governamental. Esses achados também podem ser interpretados à luz do conceito de capacidade estatal, entendido como a habilidade do Estado de formular, coordenar e implantar políticas públicas de forma consistente ao longo do tempo. Nessa perspectiva, a efetividade de políticas de caráter desenvolvimentista depende não apenas de capacidades técnico-administrativas - relacionadas à competência burocrática e à execução de ações coordenadas -, mas também de capacidades políticas, associadas à articulação de atores, à construção de consensos e à sustentação de estratégias de longo prazo em contextos institucionais complexos ^18^.

No caso das PDPs, os resultados sugerem limitações não apenas na capacidade produtiva dos laboratórios públicos, mas também na capacidade estatal de coordenação interinstitucional e de sustentação de diretrizes de política industrial em saúde, especialmente em contextos de mudança de orientação governamental. Adicionalmente, a multiplicidade de atores, instâncias decisórias e mecanismos de controle no ambiente democrático brasileiro impõe desafios à coordenação das políticas, podendo gerar descontinuidades e dificultar a implementação consistente de objetivos de transformação estrutural ^18^.

Por fim, é importante destacar que os limites identificados neste estudo não anulam os avanços produzidos pelas PDPs, mas indicam a necessidade de revisão crítica e aprimoramento do instrumento. A heterogeneidade dos resultados sugere que a política pode ser mais efetiva quando adaptada às especificidades das plataformas tecnológicas, às capacidades institucionais dos laboratórios públicos e às características das cadeias produtivas envolvidas. Nesse sentido, os achados oferecem subsídios empíricos relevantes para o redesenho de mecanismos de governança, monitoramento e avaliação das PDPs, de modo a potencializar seus efeitos sobre a capacidade produtiva nacional e a sustentabilidade do SUS.

## Considerações finais

Este estudo avaliou empiricamente os resultados das PDPs no Brasil no período de 2011 a 2024, com foco em sua contribuição para o fortalecimento da capacidade produtiva nacional de medicamentos estratégicos para o SUS. Ao privilegiar uma abordagem de avaliação formativa e um horizonte temporal ampliado, foi possível captar não apenas os resultados pontuais, mas os padrões estruturais de desempenho, os avanços e as limitações da política ao longo do tempo. Os resultados devem ser interpretados à luz do contexto institucional no período analisado, marcado por oscilações na orientação da política de compras públicas e por eventos críticos, como a pandemia de COVID-19. Em especial, a priorização de critérios de economicidade de curto prazo entre 2017 e 2022 contribuiu para a instabilidade das PDPs e para a limitação de seus efeitos estruturais, evidenciando a sensibilidade da política a mudanças de diretrizes governamentais.

Os achados indicam que as PDPs produziram avanços relevantes na internalização produtiva de determinados medicamentos, especialmente no segmento de medicamentos sintéticos, nos quais observou-se maior estabilidade produtiva e continuidade das aquisições governamentais. Esses resultados confirmam o potencial das PDPs como instrumento de articulação entre política de saúde e política industrial, quando alinhadas a rotas produtivas menos complexas e a capacidades institucionais previamente consolidadas.

Entretanto, a análise também evidenciou limites importantes da política. Embora nos anos iniciais das PDPs tenha havido maior dispersão institucional das parcerias entre diferentes laboratórios públicos, ao longo do tempo observa-se um processo de concentração em um conjunto restrito de instituições. Esse movimento parece estar associado, em parte, à maior capacidade de resposta produtiva, institucional e regulatória de determinados laboratórios, que reuniram condições para avançar nas fases mais maduras das parcerias e assegurar o abastecimento do SUS. Ainda assim, a elevada heterogeneidade institucional, a instabilidade na execução das PDPs e as dificuldades de maturação tecnológica comprometeram o alcance sistêmico da política.

Os resultados sugerem que a formalização de parcerias e a transferência de etapas produtivas finais não são suficientes, por si só, para assegurar a sustentabilidade produtiva e a autonomia tecnológica. Nesse sentido, destaca-se que a internalização observada nas PDPs concentrou-se predominantemente nas etapas finais da produção, sem implicar, na maioria dos casos, o domínio tecnológico sobre IFAs. Essa limitação representa um dos principais entraves à consolidação de uma autonomia produtiva efetiva, indicando a necessidade de políticas complementares voltadas ao fortalecimento da base farmoquímica nacional. Nesse aspecto, a efetividade das PDPs depende de condições estruturais mais amplas, como o fortalecimento da base farmoquímica e biotecnológica nacional, a estabilidade regulatória, a coordenação entre políticas públicas e o investimento contínuo em capacidades institucionais e tecnológicas dos laboratórios públicos.

Do ponto de vista das políticas públicas, os resultados do estudo indicam que os efeitos das PDPs sobre a sustentabilidade produtiva e tecnológica variam de forma significativa conforme o tipo de medicamento, o estágio de maturação da parceria e a capacidade institucional dos laboratórios envolvidos. Nesse sentido, os achados sugerem a importância de aperfeiçoar os mecanismos de monitoramento e avaliação da política, incorporando indicadores capazes de distinguir resultados intermediários - como a continuidade produtiva e o atendimento à demanda do SUS - de efeitos estruturais mais complexos, como a internalização de etapas críticas da produção. Ademais, os dados evidenciam a necessidade de maior seletividade e focalização das PDPs, especialmente nos segmentos de maior complexidade tecnológica, de modo a alinhar o desenho das parcerias à capacidade produtiva efetiva dos laboratórios públicos e aos objetivos de médio e longo prazo da política. Essas evidências reforçam que o aprimoramento das PDPs depende menos de sua expansão indiscriminada e mais do uso sistemático de informações empíricas e indicadores de desempenho para orientar ajustes incrementais na condução da política.

Em termos de contribuição inédita, este trabalho se destaca pela construção de indicadores de sustentabilidade produtiva e tecnológica capazes de capturar, de forma integrada, a maturação das parcerias e o desempenho das plataformas produtivas. Esses indicadores revelaram padrões até então pouco sistematizados: tempos prolongados de transição tecnológica, fragilidade na continuidade produtiva e altos níveis de assimetria entre instituições.

Entre as limitações do estudo, destaca-se a dependência de bases secundárias e administrativas, que podem apresentar lacunas ou assimetrias de registro. Ainda assim, a triangulação de diferentes fontes de dados e a construção de indicadores próprios permitiram mitigar parte dessas limitações e oferecer uma leitura consistente dos resultados da política ao longo do tempo.

Em síntese, as PDPs configuram um instrumento estratégico relevante, porém, insuficiente, de forma isolada, para transformar estruturalmente a dependência tecnológica do Setor Saúde no Brasil. Seu aprimoramento requer visão de longo prazo, coordenação interinstitucional e integração com políticas industriais e de inovação mais amplas, de modo a assegurar a sustentabilidade produtiva, a autonomia sanitária e o fortalecimento do SUS.

## Data Availability

As fontes de informação utilizadas no estudo estão indicadas no corpo do artigo.
